# Determinants of Access to Autism Spectrum Disorder Diagnostic Services: A Systematic Review and Meta-Analysis of Factors Associated With Diagnostic Completion, Diagnostic Pathways, and Timely Diagnosis

**DOI:** 10.7759/cureus.117972

**Published:** 2026-10-15

**Authors:** John K Muthuka, Lispah N Wanjiku, Chrisphine Onyango, Kelly Oluoch, Mary Kioko, Jacinta Maluki, Japheth Nzioki, Sara Kim

**Affiliations:** 1 Community Health and Health Promotion and Public Health Sciences, Kenya Medical Training College, Nairobi, KEN; 2 Children Residential Services, Anderson Center for Autism, New York, USA; 3 Public Health, Anderson Center for Autism, New York, USA; 4 Pharmacy, Kenya Medical Training College, Nairobi, KEN; 5 Nursing, Kenya Medical Training College, Nairobi, KEN; 6 Public Health, Kenya Medical Training College, Nairobi, KEN; 7 School of Nursing, Andrews University, Berrien Springs, USA

**Keywords:** autism spectrum disorder, diagnostic access, diagnostic completion, diagnostic pathways, healthcare disparities, health services access, systematic review, timely diagnosis

## Abstract

This systematic review and meta-analysis aimed to estimate overall access to autism spectrum disorder (ASD) diagnostic services and identify the key determinants and sources of heterogeneity across the diagnostic pathway using frequentist and Bayesian approaches. A systematic search of MEDLINE/PubMed, Embase, Scopus, Web of Science, Global Health, and grey-literature sources was conducted for studies published between January 2004 and December 2024. Studies examining ASD diagnostic completion, diagnostic pathways, diagnostic timeliness, or barriers and facilitators to diagnostic access were included. A total of 22 unique studies were synthesized, with nine studies contributing 23 quantitative effect estimates. Random-effects models using restricted maximum-likelihood (REML), Bayesian hierarchical analysis, meta-regression, subgroup analyses, and sensitivity analyses were conducted following PRISMA guidelines. The pooled random-effects estimate indicated that approximately three-quarters of observed ASD diagnostic outcomes represented successful access (74.1%, 95% CI: 65.8-81.1%). Heterogeneity was substantial (I²=88.4%) with a wide 95% prediction interval (32.8%-94.4%). Bayesian analysis produced a comparable pooled estimate (73.3%, 95% CrI: 65.3%-80.2%), with high residual heterogeneity (I²≈87.5%). By outcome domain, pooled successful outcomes were highest for diagnostic pathways (89.3%, 95% CI: 70.1%-96.7%), followed by timely diagnosis (76.3%, 95% CI: 62.9%-86.0%), and lowest for diagnostic completion (67.1%, 95% CI: 61.8%-72.0%); differences between domains were borderline significant (Qₘ=5.98, p=0.050). Timely diagnosis demonstrated particularly high heterogeneity (I²=91.2%), whereas diagnostic completion showed moderate heterogeneity (I²=40.6%). Across determinant domains, pooled estimates were highest for child developmental/neurobehavioral factors (79.5%), followed by family, socioeconomic and perceptual factors (74.2%), intervention/care-navigation factors (68.0%), and provider/clinical recognition factors (63.6%). Meta-regression identified significant variation by determinant category (Qₘ=13.48, p=0.004) and effect measure (Qₘ=7.81, p=0.020), whereas age group and geographic region were not significant moderators. Family/socioeconomic factors (B=2.703, 95% CI: 0.661-4.744; p=0.009) and child developmental/neurobehavioral factors (B=1.516, 95% CI: 0.047-2.985; p=0.043) were associated with larger effects. Publication bias assessments suggested potential small-study effects, although sensitivity analyses excluding two influential estimates produced a stable pooled outcome of 77.1% (95% CI: 71.6-81.9%), supporting robustness of the principal finding. Overall, approximately 74% of observed ASD diagnostic outcomes represented successful access, but substantial heterogeneity indicates that diagnostic access is highly context-dependent. Family and socioeconomic circumstances and child developmental characteristics emerged as important determinants, while provider-related effects were more heterogeneous. The findings support interventions spanning the entire diagnostic pathway, including developmental surveillance, screening, referral coordination, family navigation, provider capacity, specialist availability, and mechanisms to improve completion of diagnostic assessment, with further longitudinal and implementation research particularly needed in low- and middle-income countries.

## Introduction and background

Autism spectrum disorder (ASD) is a lifelong neurodevelopmental condition characterized by persistent differences in social communication and interaction, alongside restricted and repetitive patterns of behavior, interests, or activities. Although the reported global prevalence of ASD has increased substantially over the past two decades, this trend likely reflects, in part, improvements in awareness, screening, diagnostic criteria, and access to assessment services rather than changes in occurrence alone. Despite these advances, timely and equitable access to ASD diagnostic services remains a major public health challenge. Signs of ASD can often be reliably identified during the second year of life; however, the average age at diagnosis in many settings remains approximately four to five years, with substantial variation across and within countries according to health system capacity, socioeconomic circumstances, and availability of specialized diagnostic services [1,2].

Timely diagnosis provides an important entry point to evidence-based interventions, educational support, family counseling, and community services. Earlier identification facilitates earlier intervention, developmental and adaptive support, family preparedness, and more effective care planning. In contrast, delayed diagnosis may lead to missed opportunities for intervention, prolonged caregiver uncertainty, increased financial burden, and widening health inequities, particularly among underserved populations and in low-resource settings. Improving access to timely diagnostic services is therefore an important priority in child health and neurodevelopmental care [3].

Despite increasing efforts to strengthen developmental surveillance, autism screening, referral pathways, and multidisciplinary diagnostic services, substantial disparities in access persist across and within countries. Long waiting times, shortages of trained specialists, fragmented referral systems, socioeconomic disadvantage, limited caregiver awareness, stigma, and differences in healthcare organization can delay diagnosis, particularly in low- and middle-income countries. Access to ASD diagnosis is consequently shaped not only by characteristics of the child but also by interacting family, provider, and health-system factors [3,4].

Evidence suggests that determinants of access operate across multiple levels of the healthcare pathway. Child-level factors, including developmental delay, intellectual disability, language impairment, and co-occurring neurodevelopmental or psychiatric conditions, may influence recognition of ASD symptoms and referral for assessment. Family-level factors, such as parental knowledge, health-seeking behavior, socioeconomic status, and stigma, can affect care-seeking and engagement with diagnostic services. Provider-level factors, including developmental surveillance practices, responsiveness to parental concerns, use of validated screening tools, and referral decisions, may facilitate or delay diagnostic completion. At the health-system level, interventions such as family navigation programs have shown promise in reducing barriers and improving completion of diagnostic evaluations among children at risk for ASD [5].

Several systematic reviews have examined aspects of ASD diagnosis, including predictors of earlier diagnosis, global age at diagnosis, and definitions of delayed diagnosis. A recent systematic review identified clinical, social, and environmental predictors associated with earlier diagnosis but synthesized the evidence narratively because of substantial methodological heterogeneity. A systematic review and meta-analysis estimated the global age at ASD diagnosis, while a more recent review highlighted considerable inconsistency in the definition of delayed diagnosis across studies. Although these reviews have advanced understanding of diagnostic timing, they have not comprehensively quantified determinants of access across the continuum from referral and diagnostic completion to diagnostic pathways and timely diagnosis [5,6].

To our knowledge, no previous systematic review has comprehensively synthesized and quantitatively pooled evidence on the broad range of clinical, family, provider, and health-system determinants associated with access to ASD diagnostic services. Addressing this gap is important for informing interventions to reduce diagnostic inequities, strengthen referral pathways, improve diagnostic completion, and promote earlier identification of autistic children across diverse healthcare settings.

Therefore, this systematic review and meta-analysis aimed to: (1) systematically identify and synthesize quantitative evidence on factors associated with access to ASD diagnostic services; (2) evaluate determinants of diagnostic completion, diagnostic pathways, and timely diagnosis across healthcare systems; (3) quantitatively pool comparable effect estimates through meta-analysis where appropriate; and (4) explore potential sources of heterogeneity using subgroup analyses and meta-regression where sufficient data were available.

## Review

Materials and methods

Study Design and Protocol Registration

A systematic review and meta-analysis of determinants of access to ASD diagnostic services was conducted and reported in accordance with the Preferred Reporting Items for Systematic Reviews and Meta-Analyses (PRISMA) 2020 statement [7,8]. The review protocol was prospectively registered with the International Prospective Register of Systematic Reviews (PROSPERO: CRD420261326047).

Search Strategy and Study Selection

A comprehensive electronic search was conducted in MEDLINE/PubMed, Embase, Scopus, and Web of Science, supplemented by searches of grey literature sources. The search strategy combined controlled vocabulary and free-text terms related to autism spectrum disorder, diagnostic services, access, pathways, delays, determinants, barriers, and facilitators. The detailed search strategies for MEDLINE/PubMed, Embase, Scopus, and Web of Science, including the search terms, Boolean operators, and database-specific search combinations, are provided in the Appendices (Search Strategy). Studies published between January 1, 2004, and December 30, 2024, were considered in the original search. Records identified through the electronic and supplementary searches were screened by title and abstract, followed by full-text assessment of potentially eligible studies.

Eligibility Criteria

Eligibility criteria were defined a priori based on the review objectives and were applied during title/abstract screening and full-text assessment. The inclusion and exclusion criteria are summarized in Table 1.

**Table 1:** Inclusion and exclusion criteria for study selection

Criterion	Inclusion criteria	Exclusion criteria
Population	Individuals of any age undergoing or having received ASD diagnostic assessment, and caregivers or families when outcomes related to diagnostic access were reported.	Animal populations or studies not involving human participants.
Condition	Studies addressing autism spectrum disorder (ASD) diagnostic services.	Studies not related to ASD.
Outcome	Studies reporting at least one diagnostic-access outcome, including diagnostic completion, diagnostic pathways, diagnostic timeliness, age at diagnosis, diagnostic delay, or barriers and facilitators influencing access to diagnostic services.	Studies reporting only ASD prevalence, etiology, genetics, clinical characteristics, or comorbidities without a diagnostic-access outcome.
Determinants	Determinants operating at individual/child, family, socioeconomic, geographic, provider, healthcare-system, or policy levels.	Studies without information relevant to determinants of access to ASD diagnostic services.
Study design	Quantitative, qualitative, and mixed-methods studies containing sufficient information to assess determinants of diagnostic access.	Reviews, meta-analyses, editorials, commentaries, and conference abstracts without sufficient information for eligibility assessment or extraction.
Publication period	Studies published between January 1, 2004, and December 30, 2024.	Studies published outside the specified publication period.
Information availability	Studies providing sufficient information to determine eligibility and assess relevant diagnostic-access outcomes.	Studies without sufficient information to determine eligibility or assess relevant outcomes.
Quantitative synthesis	Studies reporting an effect estimate with a corresponding measure of precision, or sufficient information to calculate one, for determinants associated with diagnostic completion, diagnostic pathways, timely diagnosis, or diagnostic access.	Studies lacking sufficiently comparable outcomes or statistical information for quantitative pooling. Such studies were retained for narrative synthesis where otherwise eligible.
Language/availability	Studies for which sufficient information was available to assess eligibility and extract relevant data.	Records for which eligibility or relevant outcomes could not be adequately assessed because of insufficient information.

Data Extraction

Data were extracted independently by two reviewers (JM and KO) using a standardized data extraction form developed before the review. Extracted information included author and publication year, country and geographic region, study design, study period, healthcare setting, sample size, participant characteristics including age, diagnostic outcome, determinant examined, effect measure, effect estimate and 95% confidence interval, variables included in adjusted analyses, and information required for risk-of-bias assessment. For studies contributing to the narrative synthesis, reported barriers and facilitators were additionally extracted and categorized according to the level at which they operated, including child/individual, family, socioeconomic, geographic, healthcare-system, provider, and policy levels.

Where multiple publications appeared to use the same underlying dataset, publications were assessed for overlap to avoid treating the same study population as independent evidence. Studies were counted at the unique-study level, whereas multiple estimates reported within a study were retained where they represented distinct determinants or outcomes and met the criteria for quantitative synthesis. Disagreements between reviewers were resolved through discussion, with consultation of a third reviewer where necessary.

Risk of Bias/Methodological Quality Assessment

Two reviewers independently assessed the methodological quality of included studies using the Mixed Methods Appraisal Tool (MMAT), applying the appropriate criteria according to study design. Disagreements were resolved through discussion or consultation with a third reviewer. The appraisal findings were used to inform interpretation of the evidence and were not used as exclusion criteria.

Synthesis of Findings and Statistical Analysis

Studies that could not be quantitatively combined were synthesized narratively according to determinant domains, including child developmental and neurobehavioral factors; family, socioeconomic and perceptual factors; provider and clinical recognition factors; and intervention and care-navigation factors. Narrative findings were also considered according to three pre-specified diagnostic outcome domains: diagnostic completion, diagnostic pathways, and timely diagnosis.

Where studies reported sufficiently comparable determinants, outcomes, and effect measures, estimates were extracted and quantitatively synthesized. Eligible measures included odds ratios (ORs), risk ratios (RRs), hazard ratios (HRs), prevalence ratios (PRs), incidence rate ratios (IRRs), and other adjusted measures of association with corresponding measures of precision. Where necessary, effect estimates were transformed to the natural logarithmic scale before pooling and subsequently back transformed for interpretation.

Quantitative synthesis was conducted using JASP [9,10]. Random-effects models were estimated using restricted maximum likelihood (REML). A random-effects approach was specified a priori because substantial clinical and methodological heterogeneity was anticipated across studies with respect to populations, healthcare settings, determinants, outcome definitions, and study designs.

For the primary synthesis, pooled estimates were expressed on a common probability scale where appropriate to facilitate interpretation across the included effect estimates. The resulting pooled estimates therefore represent standardized summary estimates of successful diagnostic outcomes rather than a single directly measured probability across all studies. Between-study heterogeneity was assessed using Cochran's Q statistic, I², and τ². Ninety-five percent prediction intervals were calculated to describe the expected range of outcomes in comparable future settings.

Influence diagnostics and Baujat plots were examined to identify studies or effect estimates contributing disproportionately to the pooled estimate or observed heterogeneity. Funnel plots and residual funnel plots were examined where sufficient studies were available to assess potential small-study effects and funnel plot asymmetry.

Subgroup Analyses

Pre-specified subgroup analyses were conducted according to outcome domain, effect measure, geographic region, and determinant category. Outcome-domain analyses classified effects into three domains: diagnostic completion, representing outcomes reflecting successful completion or ascertainment of an ASD diagnosis; diagnostic pathways, representing outcomes reflecting the pathway, route, or process through which individuals accessed ASD diagnosis; and timely diagnosis, representing outcomes reflecting early, delayed, or timely receipt of an ASD diagnosis.

The subgroup analysis by determinant category classified determinants into four broad domains: Intervention and Care Navigation Factors; Provider and Clinical Recognition Factors; Child Developmental and Neurobehavioral Factors; and Family, Socioeconomic and Perceptual Factors. Omnibus subgroup tests were used to assess whether pooled estimates differed across categories. Within each subgroup, heterogeneity was assessed using Q, I², and τ² statistics. Subgroup findings were interpreted cautiously where categories contained few effect estimates.

Meta-regression Analysis

Random-effects meta-regression analyses were conducted to investigate potential sources of between-study heterogeneity. Pre-specified moderators included participant age group, determinant category, effect measure, and geographic region, where sufficient data were available. Omnibus tests were initially performed to determine whether each moderator explained significant variation in effect sizes, followed by an examination of individual regression coefficients to identify specific category differences. Multicollinearity among moderators was assessed using variance inflation factors (VIFs) where appropriate. Moderators represented by sparse or unstable categories were interpreted cautiously, and estimates were not over-interpreted where model support was limited.

Sensitivity and Robustness Analyses

Sensitivity analyses were conducted to evaluate the robustness of the pooled estimates. These included leave-one-out analyses, influence diagnostics, and exclusion of influential or outlying effect estimates identified through residual and influence analyses. Pooled estimates before and after removal of influential effect estimates were compared to determine whether individual studies materially altered the overall findings. The sensitivity analysis was also used to assess whether the observed heterogeneity and overall conclusions remained stable after exclusion of influential observations.

Bayesian Meta-analysis

To complement the frequentist analyses, a Bayesian random-effects meta-analysis was conducted using weakly informative priors. A Normal (0,1) prior was specified for the pooled log-transformed effect, and a Half-Cauchy (0,1) prior was specified for between-study heterogeneity (τ). Posterior pooled estimates, 95% credible intervals, prediction intervals, and between-study heterogeneity were estimated using Markov Chain Monte Carlo (MCMC) sampling.

Model convergence was assessed using R-hat statistics and effective sample sizes (ESS). Posterior distributions were examined to assess the precision and uncertainty of the pooled estimates. Bayesian subgroup analyses were additionally conducted for the determinant categories to complement the frequentist subgroup analyses. Bayes factors (BFs) were used to quantify the strength of evidence for inclusion of each determinant category in the meta-analytic model. Bayesian results were interpreted alongside the frequentist estimates rather than as replacements for them.

Assessment of Small-Study Effects and Publication Bias

Potential small-study effects were assessed using funnel plots and statistical tests of funnel plot asymmetry where the number of available effect estimates permitted such assessment. Because funnel plot asymmetry can arise from several mechanisms other than publication bias, including genuine between-study heterogeneity and differences in study size or methodology, evidence of asymmetry was interpreted cautiously. A fail-safe N was additionally calculated to assess the extent to which unpublished or missing null findings would be required to alter the overall statistical significance of the pooled result.

Results

Study Selection: Included Articles and Quality Assessment (Systematic Review)

The database search identified 4,899 records. After removing 537 records without associated data, 4,362 records remained for title and abstract screening. Of these, 3,800 records were excluded based on the predefined eligibility criteria, and 562 reports were sought for retrieval. A total of 112 reports could not be retrieved, leaving 450 reports for full-text eligibility assessment. Following full-text evaluation, 428 reports were excluded for predefined reasons, including inappropriate population, study design, determinant or exposure, diagnostic outcome, or insufficient methodological or quantitative information. Ultimately, 22 studies were included in the systematic review, of which nine contributed to quantitative meta-analysis, providing 23 effect estimates, while 13 contributed to the narrative synthesis only. Several studies contributed multiple effect estimates because they assessed different determinants, diagnostic outcomes, or comparison groups within the same study population. Therefore, the 23 quantitative effect estimates represented nine unique studies and were not treated as 23 independent studies (Figure 1).

**Figure 1:**
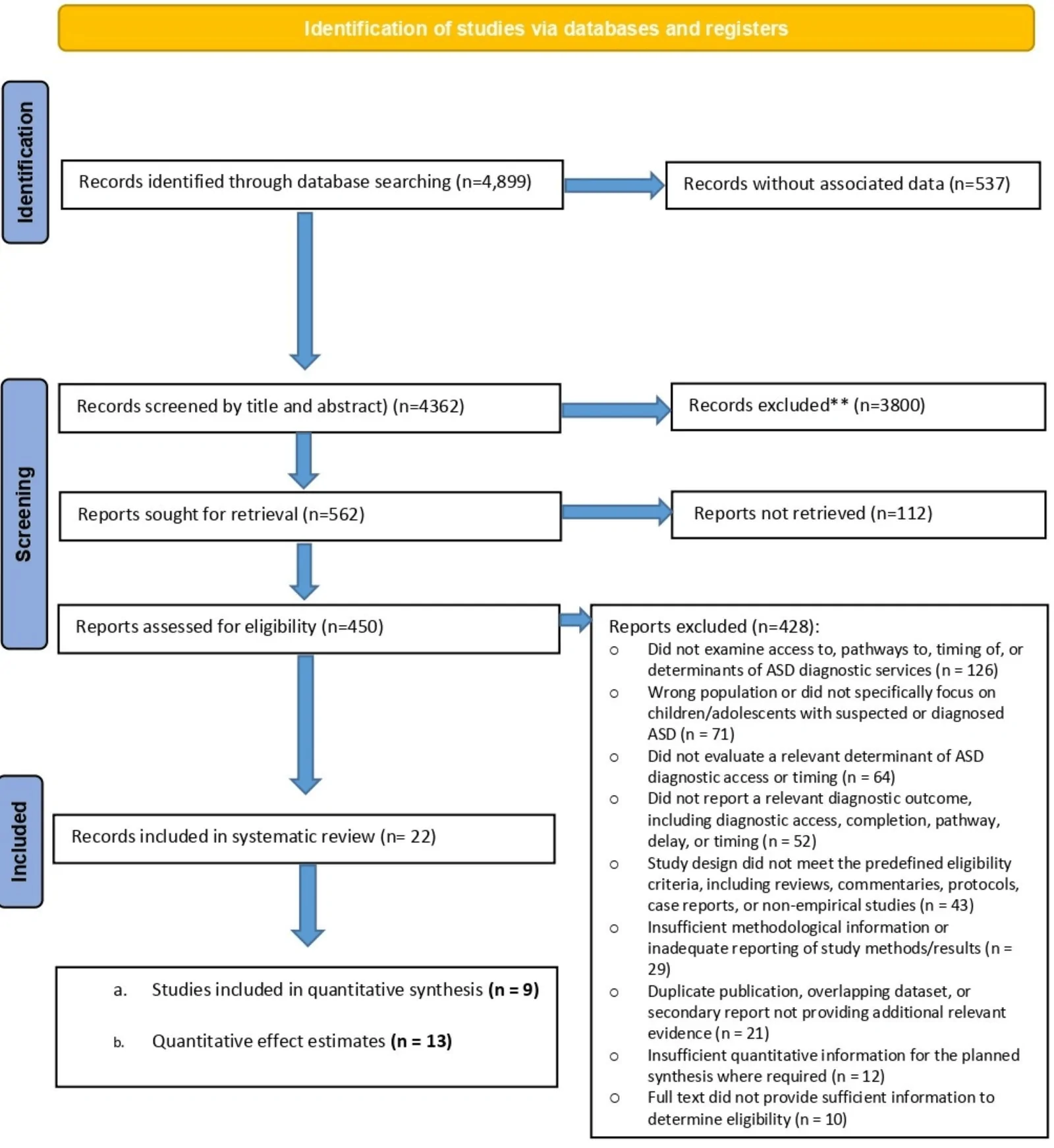
PRISMA flow diagram of study identification, screening, eligibility assessment, and inclusion **Records excluded after title and abstract screening because they did not meet the predefined eligibility criteria. PRISMA: Preferred Reporting Items for Systematic Reviews and Meta-Analyses.

The 22 included studies evaluated a broad range of determinants of access to ASD diagnostic services, including socioeconomic, geographic, individual and family, communication, screening, and health-system factors [11-32]. Nine studies contributed to the quantitative meta-analysis, providing 23 effect estimates, while 13 studies contributed to the narrative synthesis because their findings were not sufficiently comparable or lacked the statistical information required for quantitative pooling [11-32]. The quantitative studies were conducted predominantly in the United States and United Kingdom, with additional evidence from Kenya, and reported associations using hazard ratios (HRs), adjusted odds ratios (AORs), adjusted relative risk ratios (aRRRs), and adjusted prevalence ratios (APRs) [11-19]. The studies included a range of observational and other study designs and assessed outcomes related to diagnostic completion, diagnostic pathways, and timely or late diagnosis [11-32]. Overall, the evidence suggested that socioeconomic disadvantage, rural residence, limited health-system capacity and coordination, communication barriers, and missed or inconsistently implemented screening opportunities may contribute to delayed or inequitable access to ASD diagnostic services [11-32] (Tables 2, 3).

**Table 2:** Characteristics and Findings of Studies Contributing to the Narrative Synthesis Only These 13 studies contributed to the narrative synthesis only and did not provide effect estimates suitable for inclusion in the quantitative meta-analysis. Abbreviations: ASD, autism spectrum disorder; USA, United States of America.

Study ID	Study design	Region	Determinant domain	Key determinant(s)	Main diagnostic access finding
Carbone et al., 2010 [29]	Clinical/narrative review	North America (USA)	Health system	Screening implementation; provider practices; referral processes	Inconsistent implementation of developmental and ASD screening and variability in follow-up and referral contributed to missed or delayed identification.
Carbone et al., 2020 [26]	Retrospective observational study	North America (USA)	Health system/Screening	Autism screening practices; screening uptake; follow-up	Variability in screening uptake and follow-up reduced the effectiveness of routine ASD identification and referral.
DeGuzman et al., 2017 [24]	Retrospective observational/geospatial study	North America (USA)	Geographic	Rural residence; provider distribution; geographic access	Rural areas and communities with shortages of diagnostic providers had greater unmet need and reduced access to early diagnostic services.
Fountain et al., 2011 [20]	Retrospective cohort study	North America (USA)	Socioeconomic	Household income; parental education; socioeconomic status	Socioeconomic disadvantage was associated with later ASD diagnosis and reduced timely access to diagnostic assessment.
Liptak et al., 2008 [32]	Cross-sectional observational study	North America (USA)	Health system	Insurance; healthcare access; service availability	Healthcare access and financing influenced access to diagnostic and autism-related services, with disadvantaged populations experiencing greater barriers.
Maciver et al., 2025 [31]	Observational/service-evaluation study	Europe/UK	Health system	Waiting times; service organization; diagnostic pathways	Fragmented pathways, limited specialist capacity, and variation in service organization contributed to prolonged diagnostic waiting times.
Mandell et al., 2005 [21]	Retrospective observational study	North America (USA)	Socioeconomic/Geographic	Poverty; socioeconomic status; rural residence	Socioeconomic disadvantage and rural residence were associated with delayed ASD diagnosis.
Penner et al., 2018 [30]	Cross-sectional survey study	North America (USA)	Health system	Diagnostic standards; clinical contact; service coordination	Greater adherence to recommended diagnostic practices and greater clinical contact were associated with more timely diagnosis.
Pham et al., 2022 [22]	Retrospective cross-sectional observational study	North America (USA)	Socioeconomic	Socioeconomic and racial disparities	More recent evidence suggested narrowing disparities in ASD prevalence and age at diagnosis across racial/ethnic groups, although continued monitoring of equity remains important.
Pierce et al., 2019 [27]	Prospective longitudinal cohort study	North America (USA)	Screening	Early screening; developmental surveillance; repeated assessment	ASD could be reliably identified from approximately 14 months, supporting early and repeated screening and surveillance.
Prahbhjot & Singhi, 2022 [23]	Retrospective record-based observational study	Asia	Socioeconomic/Geographic	Parental education; family size; rural residence; socioeconomic status	Lower parental education, larger family size, rural residence, and socioeconomic disadvantage were associated with delayed diagnosis and reduced access.
Smith-Young et al., 2020 [25]	Qualitative study (grounded theory)	North America (Canada/USA)	Geographic	Travel distance; rurality; provider availability; referral pathways	Rural and underserved families experienced long travel distances, prolonged waits, fragmented referral pathways, and difficulties locating qualified diagnostic providers.
St. Amant et al., 2018 [28]	Cross-sectional observational study	North America (USA)	Communication	Language barriers; communication with service providers	Parents who primarily spoke a language other than English experienced barriers navigating healthcare and educational systems and accessing autism-related services.

**Table 3:** Characteristics of Studies Included in the Quantitative Synthesis Contains nine unique studies contributing 23 effect estimates. Multiple rows for the same study represent different determinants, comparisons, or diagnostic outcomes and should not be counted as separate studies. Overall, 22 unique studies were included in the review: 13 narrative-only studies and 9 quantitative studies. Abbreviations: ADHD, attention-deficit/hyperactivity disorder; AOR, adjusted odds ratio; APR, adjusted prevalence ratio; ASD, autism spectrum disorder; ASQ, Ages and Stages Questionnaire; CI, confidence interval; HR, hazard ratio; M-CHAT-R/F, Modified Checklist for Autism in Toddlers, Revised with Follow-Up; PCP, primary care provider; RCT, randomized controlled trial; UK, United Kingdom; USA, United States of America; aRRR, adjusted relative risk ratio; n, sample size.

Study ID	Region	Determinant / Exposure	Diagnostic Outcome	Effect Measure	Effect Estimate (95% CI)	Study Design	Age	Study Period
Feinberg et al., 2016 [11]	North America (USA)	Family Navigation	Diagnostic completion	HR	3.21 (1.47-6.98)	RCT	47 months	2012-2014
Feinberg, Kuhn et al., 2021 [12]	North America (USA)	Family Navigation intervention	Diagnostic ascertainment/completion	HR	1.39 (1.05-1.84)	Parallel RCT	15-27 months	2015-2018
Feinberg, Augustyn et al., 2021 [13]	North America (USA)	Modified Family Navigation vs usual care	Diagnostic completion	HR	2.57 (1.22-5.40)	RCT	24.5 months	2014-2015
Feinberg, Augustyn et al., 2021 [13]	North America (USA)	Modified Family Navigation vs previous Family Navigation	Diagnostic completion	HR	2.64 (1.31-5.30)	RCT	24.5 months	2020
Zuckerman et al., 2015 [14]	North America (USA)	All 3 proactive provider responses	≥3-year diagnostic delay	AOR	0.31 (0.17-0.54)	Cross-sectional	6-17 years	2011
Zuckerman et al., 2015 [14]	North America (USA)	All 3 passive provider responses	≥3-year diagnostic delay	AOR	3.06 (1.63-5.75)	Cross-sectional	6-17 years	2011
McNally Keehn et al., 2021 [15]	North America (USA)	PCP concern for ASD	ASD diagnosis completion	AOR	1.77 (1.21-2.57)	Prospective	18-48 months	2019-2022
McNally Keehn et al., 2021 [15]	North America (USA)	Modified Checklist for Autism in Toddlers, Revised with Follow-Up	ASD diagnosis completion	AOR	2.41 (1.45-4.02)	Prospective	18-48 months	2012-2022
McNally Keehn et al., 2021 [15]	North America (USA)	ASQ communication delay	ASD diagnosis completion	AOR	2.86 (1.65-4.97)	Prospective	18-48 months	2012-2022
McNally Keehn et al., 2021 [15]	North America (USA)	ASQ personal-social delay	ASD diagnosis completion	AOR	1.76 (1.07-2.88)	Prospective	18-48 months	2012-2022
Muthiga et al., 2025 [16]	Africa (Kenya)	Prior knowledge of neurodevelopmental disorders	Mainstream diagnostic pathway	AOR	8.17 (1.71-39.07)	Cross-sectional	108 months	2023
Muthiga et al., 2025 [16]	Africa (Kenya)	Stigma	Traditional/spiritual diagnostic pathway	AOR	8.57 (1.01-72.71)	Cross-sectional	108 months	2023
Lau et al., 2022 [17]	North America (USA)	Previous developmental delay	Late ASD diagnosis	AOR	3.46 (1.86-6.42)	Cross-sectional	144 months	2011
Lau et al., 2022 [17]	North America (USA)	Previous intellectual disability	Late ASD diagnosis	AOR	9.75 (3.00-31.60)	Prospective	24 years	2006–2016
Lau et al., 2022 [17]	North America (USA)	Previous ADHD	Late ASD diagnosis	AOR	11.07 (3.43-35.71)	Prospective	101 months	2006–2016
Lau et al., 2022 [17]	North America (USA)	Previous depression	Late ASD diagnosis	AOR	8.05 (1.07-60.03)	Prospective	-	-
Lau et al., 2022 [17]	North America (USA)	Previous behavioral/conduct disorder	Late ASD diagnosis	AOR	9.90 (3.55-27.62)	Prospective	-	-
Hosozawa et al., 2020 [18]	Europe (UK)	Low household income	Primary-school vs before-school diagnosis	aRRR	1.90 (1.03-3.53)	Prospective	0-14 years	2000-2002
Hosozawa et al., 2020 [18]	Europe (UK)	Low household income	Secondary-school vs before-school diagnosis	aRRR	2.15 (1.05-4.42)	Prospective	0-14 years	2000-2002
Hosozawa et al., 2020 [18]	Europe (UK)	No parental concerns at age 5	Primary-school vs before-school diagnosis	aRRR	3.13 (1.43-6.67)	Prospective	0-14 years	2000-2002
Hosozawa et al., 2020 [18]	Europe (UK)	No parental concerns at age 5	Secondary-school vs before-school diagnosis	aRRR	5.26 (2.33-11.11)	Prospective	0-14 years	2000-2002
Hosozawa et al., 2020 [18]	Europe (UK)	Teacher-evaluated social developmental delay	Primary-school vs before-school diagnosis	aRRR	0.50 (0.27-0.90)	Prospective	0-14 years	2002
Guthrie et al., 2019 [19]	North America (USA)	Positive M-CHAT screen	Later ASD diagnosis	APR	10.30 (7.60-14.10)	Retrospective	-	-

Risk of Methodological Quality Assessment

Methodological quality was assessed for all 22 studies, comprising 13 studies contributing to the narrative synthesis and nine contributing to the quantitative synthesis. Studies reporting multiple determinants or effect estimates were assessed once at the study level, so the 23 quantitative effect estimates were not treated as independent studies. The Mixed Methods Appraisal Tool (MMAT), 2018 version [33], was used, with design-specific criteria applied to randomized, observational, descriptive, and qualitative studies. Two reviewers independently assessed methodological quality, resolving disagreements through discussion or a third reviewer. MMAT assessments informed interpretation of the findings but were not used as exclusion criteria. Detailed study-level assessments are presented (Table 4).

**Table 4:** Methodological Quality Assessment of Included Studies Using the Mixed Methods Appraisal Tool (MMAT), 2018 Abbreviations: AOR, adjusted odds ratio; APR, adjusted prevalence ratio; aRRR, adjusted relative risk ratio; HR, hazard ratio; MMAT, Mixed Methods Appraisal Tool; N/A, not applicable; RCT, randomized controlled trial. MMAT 1–5 represent the five applicable MMAT methodological appraisal criteria. ✓, criterion met; —, criterion not applicable/not assessed.

Study ID	Study design	Effect measure	MMAT 1	MMAT 2	MMAT 3	MMAT 4	MMAT 5	MMAT score
Carbone et al., 2010 [29]	Clinical/narrative review	-	-	-	-	-	-	N/A
Carbone et al., 2020 [26]	Retrospective observational	-	✓	✓	✓	✓	-	04-May
DeGuzman et al., 2017 [24]	Retrospective observational/geospatial	-	✓	✓	✓	✓	-	04-May
Fountain et al., 2011 [20]	Retrospective cohort	-	✓	✓	✓	✓	✓	05-May
Liptak et al., 2008 [32]	Cross-sectional observational	-	✓	✓	✓	✓	-	04-May
Maciver et al., 2025 [31]	Observational/service evaluation	-	✓	✓	✓	✓	-	04-May
Mandell et al., 2005 [21]	Retrospective observational	-	✓	✓	✓	✓	✓	05-May
Penner et al., 2018 [30]	Cross-sectional survey	-	✓	✓	✓	✓	-	04-May
Pham et al., 2022 [22]	Retrospective cross-sectional observational	-	✓	✓	✓	✓	-	04-May
Pierce et al., 2019 [27]	Prospective longitudinal cohort	-	✓	✓	✓	✓	✓	05-May
Prahbhjot and Singhi, 2022 [23]	Retrospective record-based observational	-	✓	✓	✓	✓	-	04-May
Smith-Young et al., 2020 [25]	Qualitative grounded theory	-	✓	✓	✓	✓	✓	05-May
St. Amant et al., 2018 [28]	Cross-sectional observational	-	-	-	-	-	-	04-May
Feinberg et al., 2016 [11]	RCT	HR	✓	✓	✓	✓	✓	05-May
Feinberg, Augustyn et al., 2021 [12]	Parallel RCT	HR	✓	✓	✓	✓	✓	05-May
Feinberg, Kuhn et al., 2021 [13]	RCT	HR	✓	✓	✓	✓	✓	05-May
Zuckerman et al., 2015 [14]	Cross-sectional	AOR	✓	✓	✓	✓	-	04-May
McNally Keehn et al., 2021 [15]	Prospective observational	AOR	✓	✓	✓	✓	✓	05-May
Muthiga et al., 2025 [16]	Cross-sectional	AOR	✓	✓	✓	✓	-	04-May
Lau et al., 2022 [17]	Prospective observational	AOR	✓	✓	✓	✓	✓	05-May
Hosozawa et al., 2020 [18]	Prospective observational	aRRR	✓	✓	✓	✓	✓	05-May
Guthrie et al., 2019 [19]	Retrospective observational	APR	✓	✓	✓	✓	-	04-May

Socioeconomic Determinants

Five studies examined socioeconomic determinants of access to ASD diagnostic services and consistently showed that socioeconomic disadvantage was associated with delayed diagnosis and reduced access to timely diagnostic services [18,20-23]. Lower household income and poverty were repeatedly identified as important predictors of delayed ASD diagnosis, with children from economically disadvantaged families being more likely to receive diagnoses after school entry or during adolescence than children from more affluent households [18,20,21]. Similarly, lower parental educational attainment was associated with later diagnosis, whereas higher maternal or parental education facilitated earlier recognition of developmental concerns and more timely access to diagnostic assessment [20,23]. Geographic residence also interacted with socioeconomic circumstances, as children living in rural communities experienced longer delays in diagnosis than their urban counterparts, reflecting reduced availability of specialist services and diagnostic resources [21,23]. Family characteristics further influenced diagnostic timing, with larger family size associated with delayed diagnosis, particularly in rural settings [4]. Although earlier studies reported socioeconomic and racial disparities in ASD diagnosis, more recent evidence suggests that some inequities may be narrowing [22]. A large U.S. cohort study found that by 2021, the prevalence and median age at ASD diagnosis were comparable across White, Black, and Hispanic children, which may reflect improvements in screening and access to diagnostic services over time, while emphasizing the need for continued monitoring to ensure equitable access across populations [22]. Overall, the evidence indicates that socioeconomic resources - including household income, parental education, and community-level educational and healthcare resources - remain important determinants of timely access to ASD diagnostic services [18,20-23], although recent improvements suggest that some disparities may be decreasing in high-income settings.

Geographic Determinants

Three studies examined geographic determinants of access to ASD diagnostic services and consistently identified place of residence and the distribution of diagnostic resources as important influences on timely diagnosis [23-25]. Across the studies, children residing in rural or underserved communities experienced delayed ASD diagnosis and reduced access to specialized diagnostic services compared with those living in urban areas [23-25]. Rural residence was associated with later diagnosis, reflecting limited availability of specialist providers and healthcare resources [23]. Similarly, geospatial analyses identified rural counties and areas with provider shortages as having substantial unmet needs for early ASD diagnostic services, with geographic location, uneven provider distribution, and insurance-related barriers contributing to delayed diagnostic evaluations [24]. Qualitative evidence further demonstrated that families living in rural or underserved areas frequently encountered long travel distances, fragmented referral pathways, prolonged waiting times, and difficulties locating qualified diagnostic providers [25]. These geographic barriers often interacted with socioeconomic disadvantage, as families with greater financial resources were better able to travel for assessments or seek private diagnostic services, whereas lower-income families experienced additional delays [25]. Overall, the evidence indicates that geographic inequities in the availability and distribution of ASD diagnostic services remain a significant barrier to timely diagnosis, particularly for rural and underserved populations [23-25].

Health System Determinants

Six studies examined health system determinants of access to ASD diagnostic services and consistently demonstrated that healthcare organization, service delivery, referral pathways, provider capacity, and adherence to best-practice guidelines substantially influenced timely diagnosis [18,26,29-32]. Across the studies, prolonged waiting times were primarily attributed to health system factors, including fragmented referral pathways, limited specialist availability, inconsistent diagnostic practices, inadequate care coordination, and variation in service organization across healthcare settings rather than child-level characteristics [30,31]. Greater adherence to recommended diagnostic standards, standardized multidisciplinary assessment pathways, and increased clinical contact were consistently associated with shorter waiting times and more timely diagnostic evaluations [30,31].

Several studies further highlighted the importance of equitable healthcare delivery in facilitating diagnostic access. Limited healthcare resources, inadequate provider training, insufficient consultation time, and poor coordination within primary care delayed recognition of developmental concerns and hindered timely referral for specialist assessment [29]. Similarly, disparities in access to healthcare services disproportionately affected underserved populations, while enrollment in public insurance programs improved access to diagnostic and autism-related services, emphasizing the role of healthcare financing and equitable service provision in reducing diagnostic delays [32].

Evidence also demonstrated that the effectiveness of early ASD identification depends not only on the availability of screening tools but also on their consistent implementation within healthcare systems. Inconsistent uptake of routine autism screening, omission of recommended follow-up procedures, and variability in referral practices reduced the effectiveness of primary care screening programs and contributed to missed or delayed diagnoses [29]. Likewise, opportunities for early identification were frequently missed despite the presence of developmental concerns recognized by parents or teachers, suggesting deficiencies in referral pathways and coordination between healthcare and educational services. Overall, the evidence indicates that strengthening healthcare system capacity, improving coordination of multidisciplinary services, standardizing diagnostic pathways, expanding specialist availability, and ensuring timely referral following developmental concerns or positive screening are critical health system strategies for improving access to timely ASD diagnosis [29-32].

Communication Determinant

Two studies examined communication-related determinants of access to ASD diagnostic services and consistently demonstrated that effective communication between families, healthcare providers, and educational professionals facilitates timely diagnosis, whereas communication barriers contribute to delays in accessing diagnostic and support services [18,28]. Language barriers were identified as an important obstacle to navigating healthcare and educational systems, with children whose parents primarily spoke a language other than English receiving fewer autism-related support services and having reduced access to appropriate educational and disability resources, even after adjustment for demographic factors [28]. Similarly, effective communication of developmental concerns between parents, teachers, and healthcare providers was critical for timely referral and diagnosis [18]. Children whose parents expressed early concerns about their social development were more likely to receive an earlier ASD diagnosis, whereas the absence of initial parental concerns and missed opportunities to act upon concerns raised by parents or teachers contributed to delayed diagnosis, often despite socio-behavioral difficulties being evident from an early age [18]. Overall, the evidence suggests that both linguistic accessibility and effective communication across families, educational settings, and healthcare services are essential determinants of equitable and timely access to ASD diagnostic services [28].

Screening Determinants

Three studies examined screening-related determinants of access to ASD diagnostic services and consistently demonstrated that early, systematic, and well-implemented screening facilitates timely diagnosis, whereas missed screening opportunities and inconsistent implementation contribute to diagnostic delays [15,26,27]. Across the studies, early developmental surveillance and routine autism screening enabled earlier identification of children with ASD and promoted timelier referral for comprehensive diagnostic assessment. However, opportunities for early identification were frequently missed despite observable developmental concerns, particularly among children with typical-range intelligence and those from socioeconomically disadvantaged households, suggesting that existing screening and surveillance processes may be less effective for some populations [15]. The effectiveness of universal screening also depended on high screening uptake, adherence to recommended screening protocols, and timely referral following positive screening results. Inconsistent screening practices, lower screening rates among certain populations and healthcare providers, omission of recommended follow-up assessments, and variability in referral practices reduced the effectiveness of screening programs and contributed to missed or delayed diagnoses [26]. Furthermore, evidence from a prospective cohort study demonstrated that ASD can be diagnosed reliably from approximately 14 months of age, supporting the value of screening before the currently recommended 18-month age while highlighting that a single screening assessment may fail to identify a substantial proportion of children who later meet diagnostic criteria, thereby reinforcing the need for repeated developmental surveillance and ongoing re-evaluation [27]. Overall, the evidence indicates that effective implementation of universal screening programs, combined with repeated developmental surveillance and timely referral pathways, is essential for improving early identification and reducing delays in ASD diagnosis [15,26,27].

Pooled Effect and Heterogeneity of Determinants of Access to ASD Diagnostic Services

The quantitative synthesis included nine unique studies from the United States, United Kingdom, and Kenya, contributing 23 quantitative effect estimates [11-19]. Because the included studies reported different effect measures, the reported estimates were harmonized using their corresponding effect sizes and standard errors for quantitative synthesis. The random-effects meta-analysis yielded a pooled effect of 74.1% (95% CI: 65.8%-81.1%). The pooled effect was statistically significant (t(22)=5.46, p<0.001). Substantial between-study heterogeneity was observed (Qₑ (22)=209.95, p<0.001), with a between-study standard deviation of τ=0.832 (95% CI: 0.587-1.238) and between-study variance of τ²=0.691 (95% CI: 0.344-1.533). The I² was 88.4% (95% CI: 79.1%-94.4%), indicating that most of the observed variability was attributable to differences between studies rather than sampling error. The 95% prediction interval ranged from 32.8% to 94.4%, indicating substantial variation in the expected diagnostic access outcome across different populations and settings (Table 5 and Figure 2).

**Table 5:** Meta-Analytic Summary of Determinants of Access to ASD Diagnostic Services Abbreviations: CI, confidence interval; H², heterogeneity statistic; I², I-squared heterogeneity statistic; PI, prediction interval; τ, between-study standard deviation; τ², between-study variance.

Parameter	Estimate	95% CI	95% PI
Back-transformed pooled proportion	74.10%	65.8%-81.1%	32.8%–94.4%
Between-study standard deviation (τ)	0.832	0.587-1.238	-
Between-study variance (τ²)	0.691	0.344-1.533	-
I² (heterogeneity)	88.40%	79.1%-94.4%	-
H² (heterogeneity)	8.588	4.777-17.825	-

**Figure 2:**
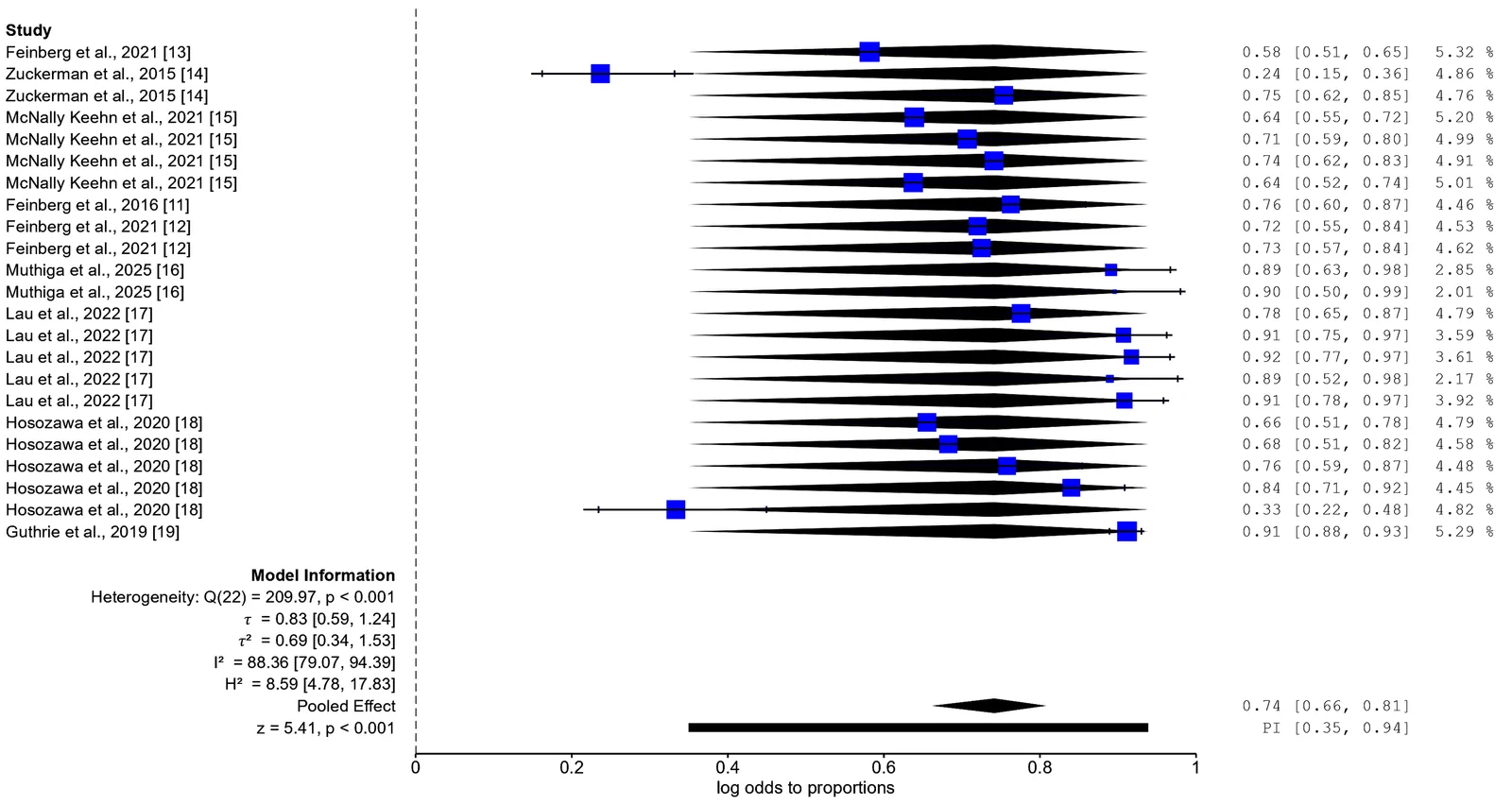
Forest plot of pooled diagnostic access outcome Random-effects meta-analysis showing a pooled effect of 74% (95% CI: 66%-81 %, p<0.001), with substantial heterogeneity (I²=88.3%). Study reference numbers in the forest plot correspond to the studies included in the meta-analysis. Some studies contribute multiple estimates and therefore appear more than once.

Stratified Analyses by Outcome Domain

Stratified analyses showed that the pooled proportion of successful diagnostic outcomes differed borderline significantly across the three outcome domains (Qₘ(2)=5.98, p=.050). Diagnostic pathways had the highest pooled proportion of 0.893 (95% CI: 0.701-0.967), followed by timely diagnosis at 0.763 (95% CI: 0.629-0.860), while diagnostic completion had the lowest pooled proportion at 0.671 (95% CI: 0.618-0.720). In simple terms, people were most likely to successfully navigate a diagnostic pathway, somewhat less likely to receive a timely diagnosis, and least likely to complete the diagnostic process. This suggests that the likelihood of a successful diagnostic outcome may vary depending on which aspect of the diagnostic process is considered. The pooled effects were statistically significant for all three outcome domains: diagnostic completion (z=6.02, p<.001), timely diagnosis (z=3.57, p<.001), and diagnostic pathways (z=3.28, p=.001). Heterogeneity differed substantially across domains. Diagnostic completion showed moderate heterogeneity (I²=40.6%, Qₑ (7)=11.13, p=.133), while diagnostic pathways showed no observed heterogeneity (I²=0%, Qₑ(1)=0.00, p=.972). In contrast, timely diagnosis demonstrated very high heterogeneity (I²=91.2%, Qₑ(12)=172.43, p<.001), indicating considerable variation in findings across studies, particularly for timely diagnosis. The prediction intervals further illustrate this difference. The estimated proportion for diagnostic completion would be expected to fall between 0.561 and 0.765 in a comparable future setting, whereas the prediction interval for timely diagnosis was considerably wider (0.256-0.968), reflecting substantial between-study variability. For diagnostic pathways, the prediction interval was 0.701-0.967, although this estimate should be interpreted cautiously because this subgroup was based on only two effect estimates from the same study. Overall, the stratified analysis suggests that outcome domain may partly explain differences in diagnostic outcomes, with the highest pooled proportion observed for diagnostic pathways and the lowest for diagnostic completion. However, the substantial heterogeneity for timely diagnosis indicates that findings in this domain vary considerably across studies, populations, and/or definitions of timely diagnosis. The borderline subgroup test (p=.050) suggests that these differences are suggestive rather than definitive evidence that outcome domain influences the likelihood of successful access to ASD diagnostic services (Table 6).

**Table 6:** Stratified Pooled Effects and Heterogeneity by Outcome Domain CI=Confidence interval; PI=prediction interval; I² = percentage of variability due to between-study heterogeneity; Qₑ=test of residual heterogeneity within subgroup; Qₘ=test of differences between subgroups. The pooled effect is transformed from log odds to proportions. Diagnostic pathways should be interpreted cautiously because this subgroup was based on only two effect estimates from the same study.

Outcome domain	Pooled proportion	95% CI	95% PI	I² (%)	Qₑ	p-value
Diagnostic completion	0.671	0.618-0.720	0.561-0.765	40.6	11.13	0.133
Timely diagnosis	0.763	0.629-0.860	0.256-0.968	91.2	172.43	< .001
Diagnostic pathways	0.893	0.701-0.967	0.701-0.967	0	0	0.972
Test for subgroup differences	-	-	-	-	Qₘ(2)=5.98	0.05

Bayesian Pooled Meta-Analysis Results and Diagnostics

The Bayesian meta-analysis shows a high pooled diagnostic access outcome, with a pooled mean of 73.3% (95% CI: 65.3%-80.2%) and an identical median, indicating a symmetric posterior distribution. Substantial between-study variability was observed (I²=87.5%; τ=0.833), while the prediction interval (32.8%-93.9%) indicates considerable variation in expected diagnostic access across different populations and settings. The MCMC diagnostics indicate reliable estimation, with low MCMC error, large effective sample sizes (ESS=15,003 for μ; 8,786 for τ), and R-hat values of 1.000, supporting model convergence. Overall, the Bayesian findings closely corroborate the frequentist meta-analysis, which yielded a pooled outcome of 74.1% (95% CI: 65.8%-81.1%), with both approaches indicating substantial heterogeneity across studies (Table 7 and Figure 4).

**Table 7:** Bayesian Meta-Analytic Estimates for Diagnostic Access CI=Credible interval; PI=prediction interval; ESS=effective sample size; R-hat=convergence diagnostic. The pooled Bayesian effect estimate (73.3%) closely agrees with the frequentist pooled estimate (74.1%), supporting consistency between the two analytical approaches. Dashes (-) indicate that the measure is not applicable.

Parameter	Estimate	95% CI	95% PI / Range
Pooled effect (μ, mean)	73.30%	(65.3%, 80.2%)	(32.8%, 93.9%)
μ (median)	73.30%	-	-
Between-study heterogeneity			
τ (SD)	0.833	(0.579, 1.198)	-
τ² (variance)	0.719	(0.335, 1.436)	-
I² (%)	87.48	(78.62, 94.03)	-
H²	8.884	(4.678, 16.755)	-
MCMC diagnostics			
MCMC error (μ)	0.002	-	-
MCMC error/SD (μ)	0.008	-	-
ESS (μ)	15,003	-	-
R-hat (μ)	1	-	-
MCMC error (τ)	0.002	-	-
MCMC error/SD (τ)	0.011	-	-
ESS (τ)	8,786	-	-
R-hat (τ)	1	-	-

**Figure 3:**
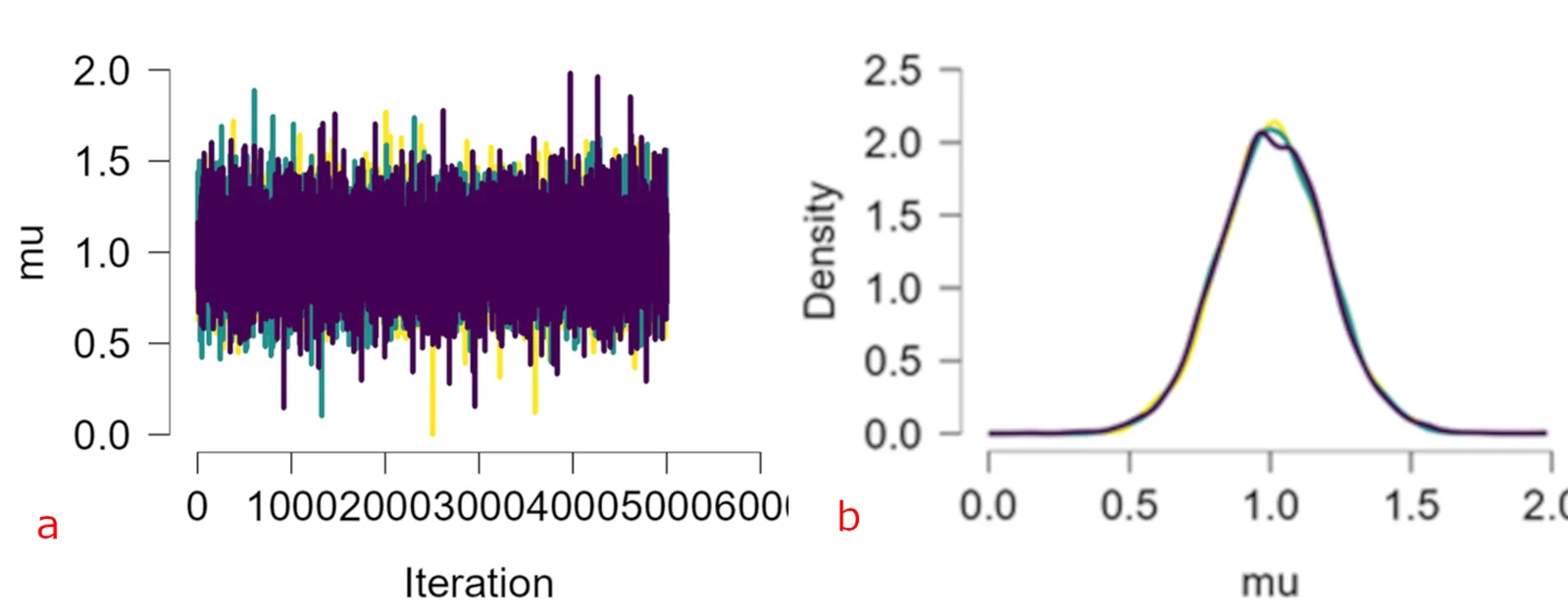
MCMC diagnostic plots for the model parameters: (a) trace plots across multiple chains and (b) autocorrelation function. The trace plots show well-mixed, stable chains, indicating good convergence, while autocorrelation decreases rapidly, indicating efficient sampling and low dependence between successive draws. MCMC: Markov Chain Monte Carlo.

Assessment of Small-Study Effects and Publication Bias

Funnel plot asymmetry was assessed using three complementary tests. The meta-regression test showed significant asymmetry (z=2.465, p=.014), and the rank correlation test was also significant (τ=0.462, p=.002), suggesting possible small-study effects. However, the weighted regression test was not statistically significant (t=0.677, df=21, p=.506), providing no evidence of asymmetry by this method. Thus, the findings provide mixed evidence of funnel plot asymmetry, with two of three tests indicating potential small-study effects. The Rosenthal fail-safe N was 1,723, indicating that a large number of additional null studies would be required to reduce the overall finding to non-significance. Overall, the results suggest some evidence of possible small-study effects, but do not provide consistent evidence of publication bias across all tests (Table 8 and Figure 4).

**Table 8:** Assessment of Small-Study Effects and Publication Bias df=Degrees of freedom; μ=limit estimate; CI=confidence interval. Significant meta-regression and rank correlation tests suggest possible small-study effects, whereas the weighted regression test was not significant. The Rosenthal fail-safe N indicates that 1,723 additional null studies would be required to reduce the overall finding to non-significance.

Method	Statistic	df	p-value	Estimate (μ)	95% CI
Meta-regression test	z=2.465	-	0.014	0.194	(-0.567, 0.954)
Weighted regression test	t=0.677	21	0.506	0.651	(-0.258, 1.560)
Rank correlation test	τ=0.462	-	0.002	-	-
Rosenthal fail-safe N	1,723		-	-	-

**Figure 4:**
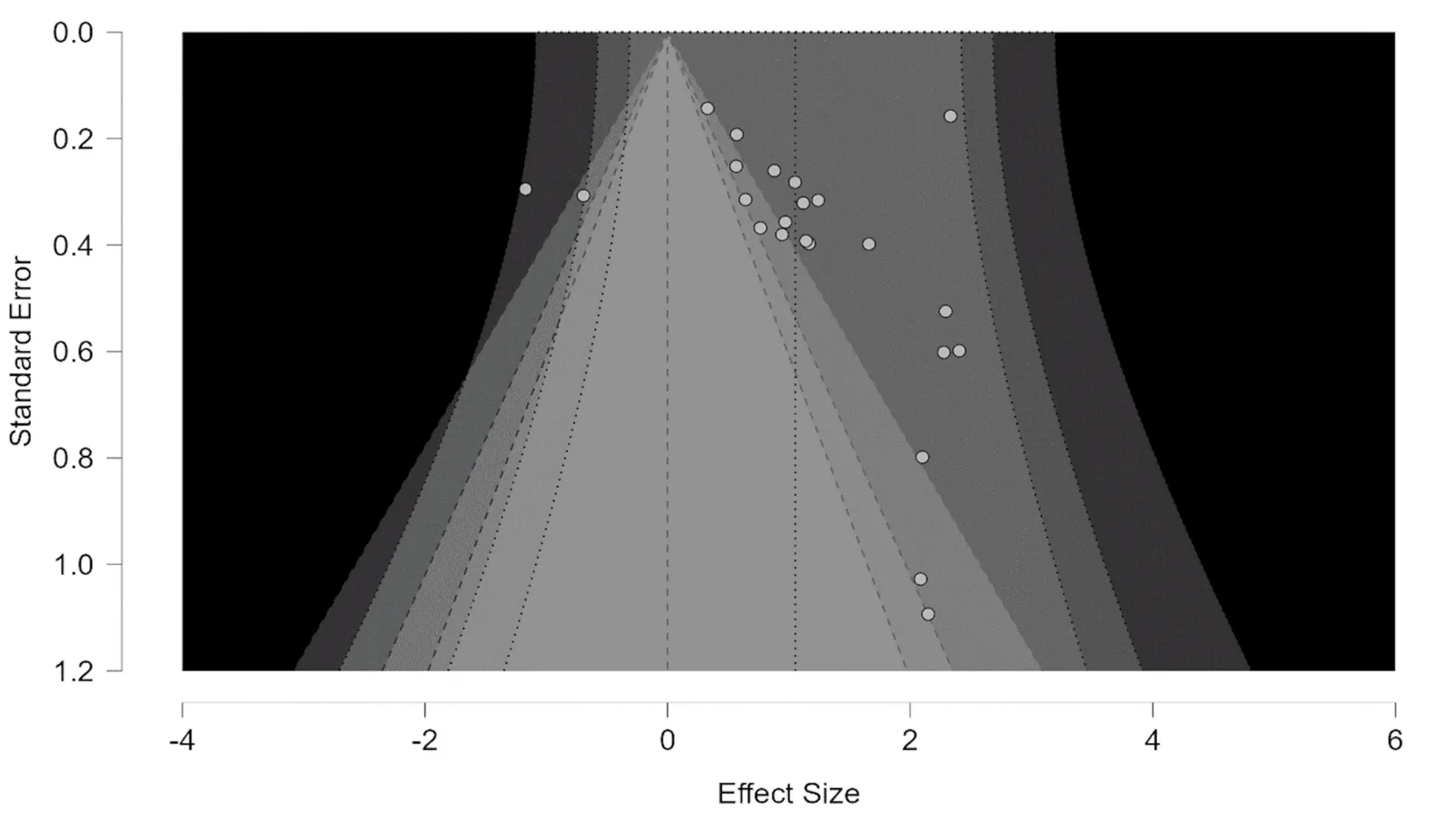
Funnel plot for assessment of small-study effects Funnel plot showing the distribution of 23 effect estimates around the pooled effect, with evidence of asymmetry suggesting possible small-study effects. Meta-regression (p=.014) and rank correlation (p=.002) tests supported asymmetry, whereas weighted regression was not significant (p=.506).

Assessment of Funnel Plot Asymmetry and the Impact of Trim and Fill Adjustment on Effect Size Estimates

The trim-and-fill analysis identified seven potentially missing estimates among the 23 effect estimates, producing an adjusted pooled effect of 0.684 (95% CI: 0.277-1.091, p<.001) and an adjusted heterogeneity estimate (τ) of 1.024 (95% CI: 0.771-1.491, p<.001). The adjusted pooled effect remained statistically significant, indicating that the overall finding persists after accounting for potential missing estimates. However, the wide confidence interval and increased τ suggest considerable uncertainty and substantial between-study variability. Overall, the trim-and-fill results provide evidence that potential small-study effects may influence the observed estimates, but they do not eliminate the evidence of a positive pooled effect (Table 9 and Figure 5).

**Table 9:** Trim and Fill Parameter Estimates μ=Pooled effect; τ=between-study standard deviation; CI=confidence interval. The trim-and-fill procedure identified seven potentially missing estimates and produced statistically significant adjusted estimates for both the pooled effect and between-study heterogeneity.

Parameter	Missing Estimates	Adjusted Estimate	Lower 95% CI	Upper 95% CI	p
Pooled effect (μ)	7	0.684	0.277	1.091	< .001
Heterogeneity (τ)	7	1.024	0.771	1.491	< .001

**Figure 5:**
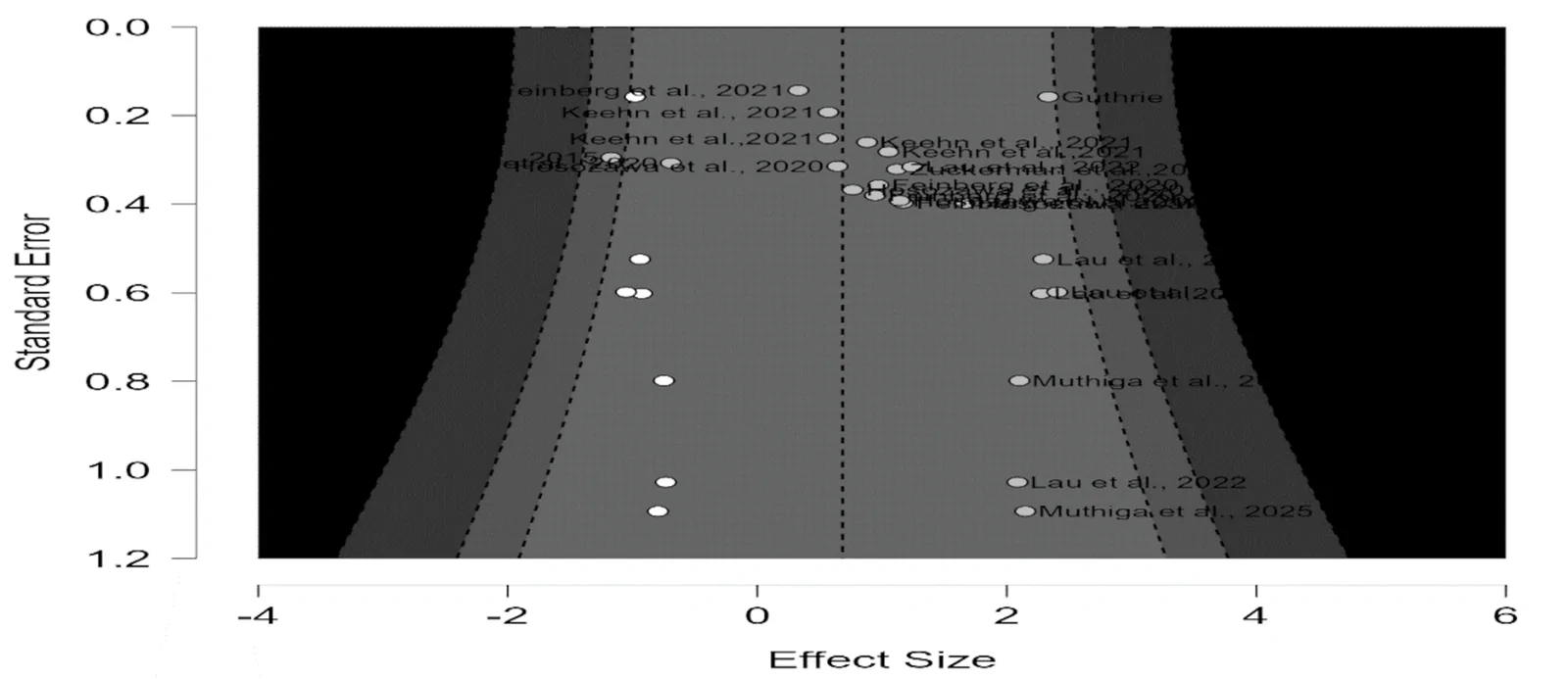
Trim-and-fill analysis showing the impact of potentially missing estimates on the pooled effect The analysis identified seven potentially missing estimates and produced an adjusted pooled effect of 0.684 (95% CI: 0.277-1.091, p<.001), indicating that the overall effect remained statistically significant after adjustment.

Sensitivity Analysis and Robustness of Findings

After conducting a sensitivity analysis involving the removal of two outlier effect estimates from two studies ((Study 1); (Study 2)), identified from the residual funnel plot (Figure 7a), the overall pooled effect remained robust. The sensitivity analysis yielded a pooled effect estimate of 0.771 (95% CI: 0.716-0.819), corresponding to an estimated probability of a positive outcome of approximately 77% (95% PI: 52.5%-91.1%). Heterogeneity remained statistically significant (Qₑ(20)=125.41, p<.001), with between-study variance of τ²=0.302 (τ=0.549). Overall, the findings indicate that removing the two outlier effects had limited impact on the pooled estimate, supporting the robustness of the original findings. However, the remaining prediction interval indicates continued variation in the expected diagnostic access outcome across studies and settings (Table 10).

**Table 10:** Sensitivity Analysis of Pooled Diagnostic Access Outcome (Outlier Effects Removed) Two outlier effects from two studies [14], [18] were removed. df=Degree of freedom; 95% CI=confidence interval; 95% PI=prediction interval; τ=between-study standard deviation; τ²=between-study variance.

Statistic	Value	95% CI	95% PI
Pooled effect (logit)	0.771	0.716-0.819	0.525-0.911
τ (between-study SD)	0.549	0.343-0.869	-
τ² (between-study variance)	0.302	0.118-0.754	-
Qₑ (df=20)	125.41	-	-
p-value (Qₑ)	< .001	-	-
Pooled effect z	8.18	-	-
p-value (z)	< .001	-	-

**Figure 6:**
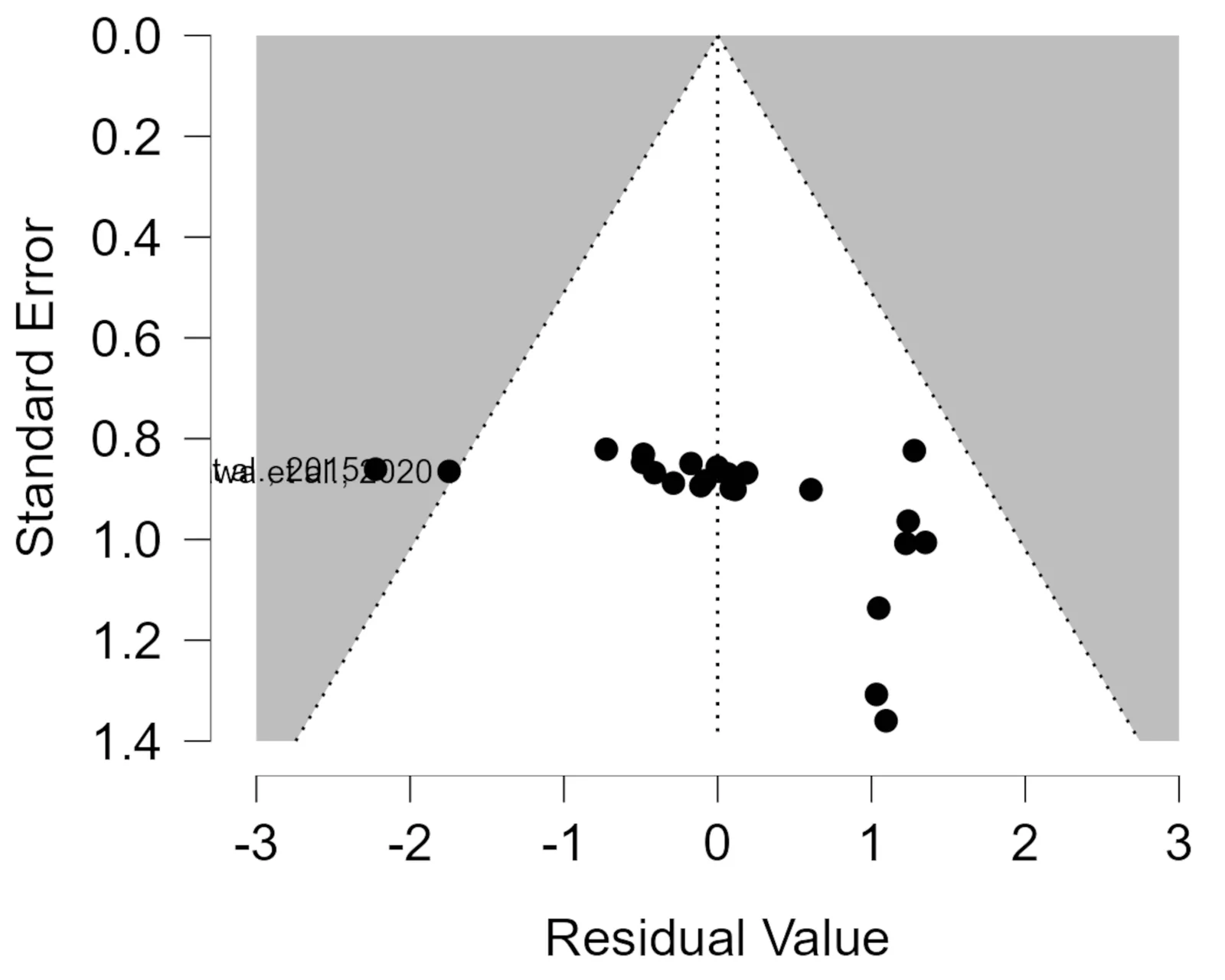
Residual funnel plot identifying outlying effect estimates Residual funnel plot showing two outlying effect estimates contributed by [14,18]. These outliers were removed in the sensitivity analysis to assess the robustness of the pooled diagnostic access outcome.

**Figure 7:**
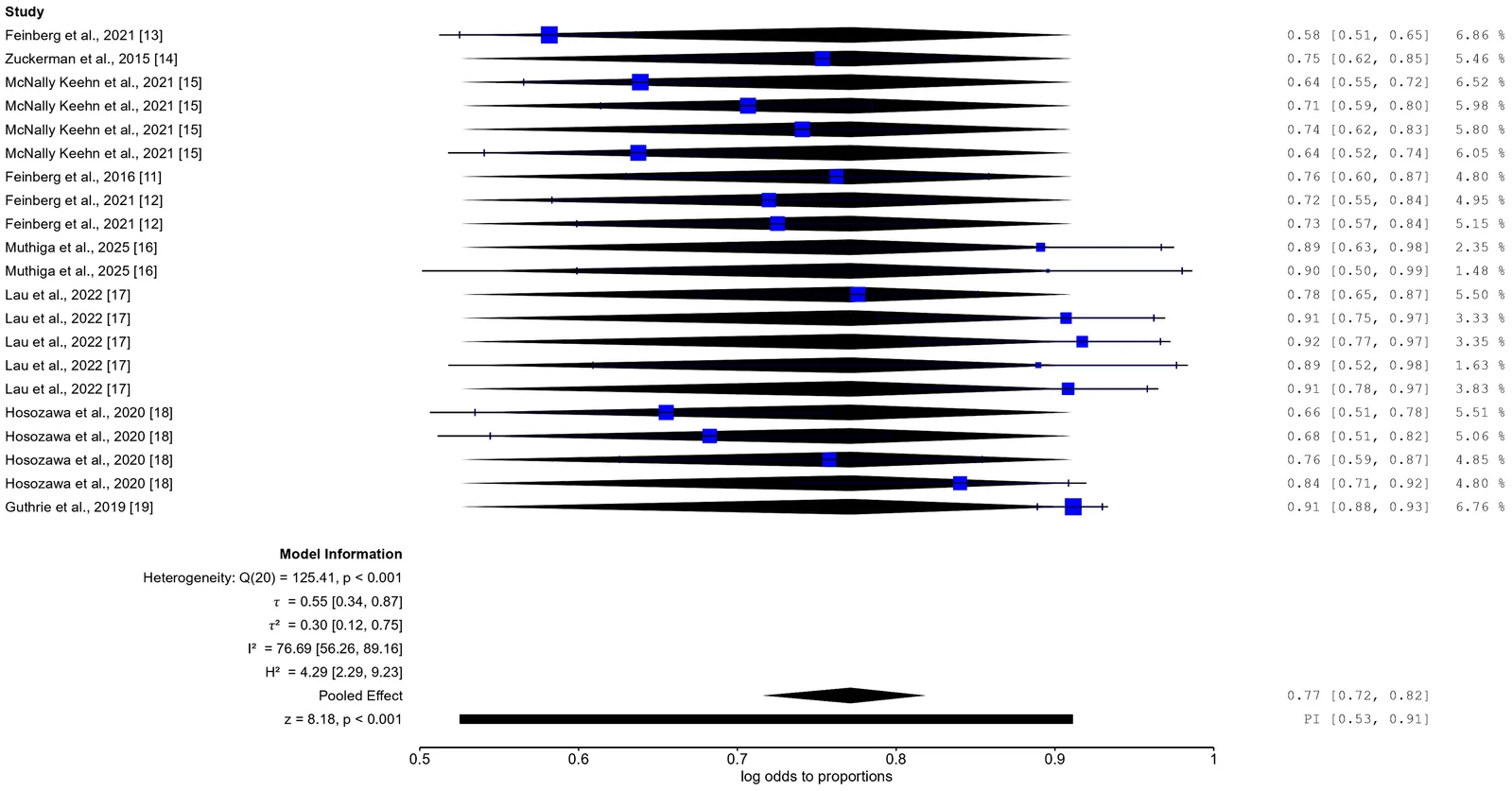
Forest plot of sensitivity analysis after removal of outlier effects Forest plot showing the pooled diagnostic access outcome after excluding outlier effects from [14], [18], with a pooled effect of 77.1% (95% CI: 72%–82%) and substantial residual heterogeneity.

Frequentist Subgroup Analysis

*By effect measure*: The subgroup analysis showed statistically significant pooled effects for HR and AOR, but not for aRRR, with no evidence that the pooled effects differed significantly across effect-measure subgroups. Studies reporting HRs yielded a pooled estimate of 0.680 (95% CI: 0.576-0.768; 95% PI: 0.490-0.824; p<.001), with low and non-significant heterogeneity (Qₑ(3)=7.23, p=.065), although the prediction interval indicates some variation across settings. Studies reporting AORs yielded a pooled estimate of 0.768 (95% CI: 0.650-0.855; 95% PI: 0.327-0.957; p<.001), but showed substantial heterogeneity (Qₑ(12)=77.80, p<.001), with τ=0.933 and τ²=0.871. Studies reporting aRRRs yielded a pooled estimate of 0.664 (95% CI: 0.476-0.811; 95% PI: 0.251-0.921), but the pooled effect was not statistically significant (z=1.71, p=.087), with substantial heterogeneity (Qₑ(4)=26.85, p<.001, τ=0.813 and τ²=0.660). The APR subgroup could not be pooled because fewer than two estimates were available. Importantly, the test for subgroup differences was not statistically significant (Qₘ(2)=1.71, p=.425), indicating no evidence that the pooled effects differed systematically according to effect measure. Overall, the subgroup findings demonstrate statistically significant effects for HR and AOR, while aRRR did not reach statistical significance, with considerable heterogeneity particularly among AOR and aRRR estimates (Table 11 and Figure 8).

**Table 11:** Subgroup Meta-Analytic Effect Estimates by Effect Measure HR=Hazard ratio; AOR=adjusted odds ratio; aRRR=adjusted relative risk ratio; APR=adjusted prevalence ratio; CI=confidence interval; PI=prediction interval. Effect estimates are retained on their original effect-measure scales because HR, AOR, aRRR, and APR are not directly comparable as proportions. The APR subgroup could not be estimated because fewer than two estimates were available. Subgroup differences were not statistically significant, Qₘ(2)=1.71, p=.425.

Effect measure	Pooled estimate	95% CI	95% PI	p-value	Heterogeneity
HR	0.68	(0.576, 0.768)	(0.490, 0.824)	< .001	Qₑ(3)=7.23, p=.065
AOR	0.77	(0.650, 0.855)	(0.327, 0.957)	< .001	Qₑ(12)=77.80, p<.001
aRRR	0.66	(0.476, 0.811)	(0.251, 0.921)	0.087	Qₑ(4)=26.85, p<.001
APR	-	-	-	-	<2 estimates
Subgroup differences	Qₘ(2)=1.71	-	-	0.425	-

**Figure 8:**
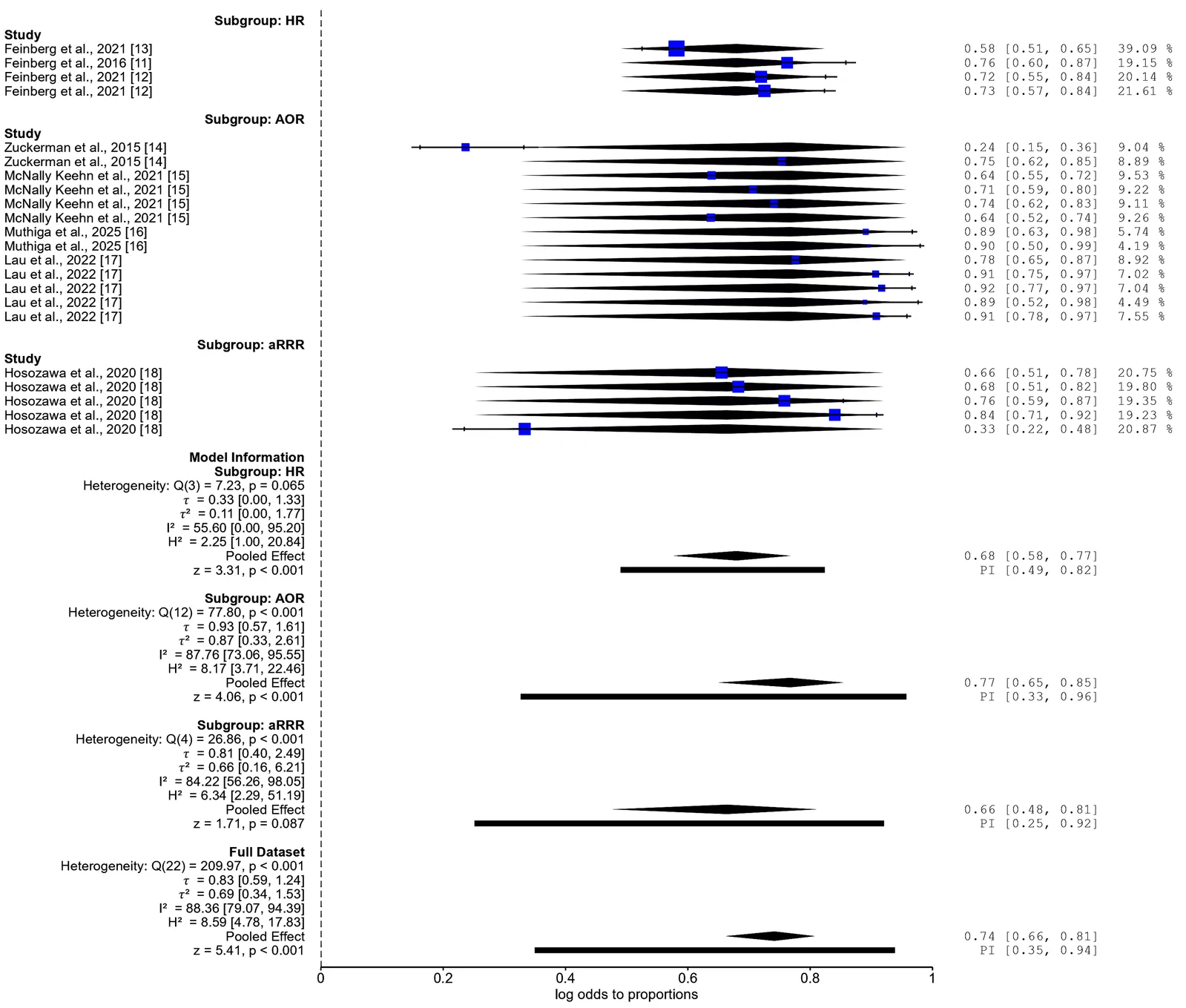
Forest plot of subgroup analysis by effect measure Forest plot showing pooled effects for HR, AOR, and aRRR estimates, with statistically significant pooled effects for HR and AOR but not aRRR. No significant differences were observed between effect-measure subgroups (Qₘ (2) = 1.71, p = .425).

*By geographic region: *The subgroup analysis showed variation in the pooled diagnostic access outcome across geographic regions, although the differences between regions were not statistically significant. North America (USA) had a pooled effect of 75.1% (95% CI: 65.6%-82.7%; 95% PI: 34.9%-94.4%; p<.001), with substantial between-study heterogeneity (Qₑ(15)=173.64, p<.001, τ=0.851 and τ²=0.724). Africa (Kenya) showed the highest pooled effect at 89.3% (95% CI: 70.1%-96.7%; 95% PI: 70.1%-96.7%; p=.001), with no observed between-study heterogeneity (τ=0.000; τ²=0.000), although the subgroup comprised only two estimates. Europe (United Kingdom) had a pooled effect of 66.4% (95% CI: 47.6%-81.1%; 95% PI: 25.1%-92.1%; p=.087) and substantial heterogeneity (Qₑ(4)= 26.85, p<.001, τ=0.813 and τ²=0.660). The test for subgroup differences was not statistically significant (Qₘ(2)= 3.61, p=.165), indicating that geographic region did not significantly explain differences in pooled diagnostic access outcomes. Overall, although the pooled outcome was highest in Kenya and lowest in the United Kingdom, the wide prediction intervals and substantial heterogeneity in the USA and UK indicate considerable variability within regions (Table 12).

**Table 12:** Subgroup Meta-Analytic Effect Estimates by Geographic Region CI=Confidence interval; PI=prediction interval; τ=between-study standard deviation. Effect estimates are presented as proportions after log-odds-to-proportion transformation. The Kenya subgroup had no observed heterogeneity, but this estimate should be interpreted cautiously given the small number of contributing estimates.

Geographic region	Pooled effect	95% CI	95% PI	p-value	Heterogeneity
North America (USA)	75.10%	(65.6%, 82.7%)	(34.9%, 94.4%)	< .001	Qₑ (15)=173.64, p<.001; τ=0.851
Africa (Kenya)	89.30%	(70.1%, 96.7%)	(70.1%, 96.7%)	0.001	Qₑ(1)=0.00, p=.972; τ=0.000
Europe (UK)	66.40%	(47.6%, 81.1%	(25.1%, 92.1%)	0.087	Qₑ(4)=26.85, p<.001; τ=0.813
Subgroup differences	-	-	-	Qₘ(2)=3.61, p=.165	-

Determinant Category

The subgroup analysis showed that three of the four determinant categories were significantly associated with ASD diagnostic access, although the magnitude of effects varied across categories. Intervention and Care Navigation Factors had a pooled effect of 68.0% (95% CI: 57.6%-76.8%; 95% PI: 49.0%-82.4%; p<.001), with relatively low and non-significant heterogeneity (Qₑ(3)=7.23, p=.065). Provider and Clinical Recognition Factors had a pooled effect of 63.6% (95% CI: 32.3%-86.4%; 95% PI: 9.9%-96.5%), but this effect was not statistically significant (p=.400) and showed substantial heterogeneity (Qₑ(3)=38.42, p<.001; τ=1.249). Child Developmental and Neurobehavioral Factors demonstrated the highest pooled effect at 79.5% (95% CI: 66.8%-88.2%; 95% PI: 34.5%-96.6%; p<.001), with substantial heterogeneity (Qₑ(9)=106.35, p<.001; τ=0.962). Family, Socioeconomic and Perceptual Factors also showed a significant pooled effect of 74.2% (95% CI: 65.0%-81.7%; 95% PI: 59.1%-85.2%; p<.001), with relatively low heterogeneity (Qₑ(4)=5.67, p=.225; τ=0.272). However, the test for subgroup differences was not statistically significant, Qₘ(3)=2.80, p=.424, indicating that the apparent differences in pooled effects across determinant categories were not statistically distinguishable. Overall, the findings suggest that child developmental/neurobehavioral and family-level factors were associated with the highest pooled diagnostic access outcomes, while provider/clinical recognition factors showed the greatest variability and did not demonstrate a statistically significant pooled association. The significant heterogeneity within the provider and child developmental/neurobehavioral categories indicates that individual determinants within these broad domains may operate differently across populations and settings (Table 13).

**Table 13:** Subgroup Meta-Analytic Effect Estimates by Determinant Category CI=Confidence interval; PI=prediction interval; τ=between-study standard deviation; τ²=between-study variance. Effect estimates are presented as proportions after log-odds-to-proportion transformation. Although pooled effects differed numerically across determinant categories, the test for subgroup differences was not statistically significant.

Determinant category	Pooled effect	95% CI	95% PI	p-value	Heterogeneity
Intervention and Care Navigation Factors	68.00%	(57.6%, 76.8%)	(49.0%, 82.4%)	< .001	Qₑ(3)=7.23, p=.065
Provider and Clinical Recognition Factors	63.60%	(32.3%, 86.4%)	(9.9%, 96.5%)	0.4	Qₑ(3)=38.42, p<.001
Child Developmental and Neurobehavioral Factors	79.50%	(66.8%, 88.2%)	(34.5%, 96.6%)	< .001	Qₑ(9)=106.35, p<.001
Family, Socioeconomic and Perceptual Factors	74.20%	(65.0%, 81.7%)	(59.1%, 85.2%)	< .001	Qₑ(4)=5.67, p=.225
Subgroup differences	-	-	-	Qₘ(3)=2.80, p=.424	-

Bayesian Subgroup Meta-Analysis by Determinant Category

The Bayesian subgroup meta-analysis demonstrated positive pooled effects across all four determinant categories, although the strength of Bayesian evidence and the degree of between-study heterogeneity varied substantially. Intervention and Care Navigation Factors showed a pooled effect of 64.3% (95% credible interval [CrI]: 50.0%-74.7%; 95% prediction interval (PI): 43.9%-80.7%), with strong evidence for inclusion (BF=25.60) and relatively low heterogeneity (I²=27.5%). Provider and Clinical Recognition Factors showed a pooled effect of 53.7% (95% CrI: 38.9%-76.2%; 95% PI: 7.1%-94.9%), but evidence for inclusion was weak (BF=0.684), while heterogeneity was very high (I²=89.5%), indicating considerable uncertainty and variability across studies. Child Developmental and Neurobehavioral Factors demonstrated a high pooled effect of 76.7% (95% CrI: 58.0%-86.1%; 95% PI: 25.8%-96.5%), with strong evidence for inclusion (BF=53.76) and substantial heterogeneity (I²=88.9%). Family, Socioeconomic and Perceptual Factors also showed a high pooled effect of 72.9% (95% CrI: 64.2%-80.0%; 95% PI: 60.3%-82.4%), with very strong evidence for inclusion (BF=226.32) and low heterogeneity (I²=10.8%). Thus, the Bayesian analysis provides the strongest evidence for Family, Socioeconomic and Perceptual Factors, followed by Child Developmental and Neurobehavioral Factors and Intervention and Care Navigation Factors, whereas evidence for Provider and Clinical Recognition Factors was inconclusive. Importantly, these findings closely correspond with the frequentist analysis: the frequentist pooled effects were 68.0%, 63.6%, 79.5%, and 74.2%, respectively, while the Bayesian estimates were 64.3%, 53.7%, 76.7%, and 72.9%. Both approaches therefore identify child developmental/neurobehavioral and family-related factors as having the highest pooled outcomes, while provider/clinical recognition factors show the greatest uncertainty and heterogeneity. Overall, the Bayesian findings reinforce the frequentist results while providing additional information on the strength of evidence and uncertainty within each determinant category (Table 14 and Figure 9).

**Table 14:** Bayesian Subgroup Meta-Analysis by Determinant Category CrI=Credible interval; PI=prediction interval; BF=inclusion Bayes factor; I²=percentage of total variation attributable to between-study heterogeneity. Effect estimates are presented as proportions after log-odds-to-proportion transformation. Bayes factors >1 support inclusion of the pooled effect, whereas values <1 provide evidence against inclusion. The very large BF for Family, Socioeconomic and Perceptual Factors should be interpreted cautiously because the software notes potential computational instability for very large Bayes factors.

Determinant category	Effect (Mean)	95% CrI	95% PI	BF	I² (%)	Interpretation
Intervention and Care Navigation Factors	0.643	0.500-0.747	0.439-0.807	25.6	27.5	Positive effect; strong evidence; low heterogeneity
Provider and Clinical Recognition Factors	0.537	0.389-0.762	0.071-0.949	0.684	89.5	Positive but uncertain effect; weak evidence; substantial heterogeneity
Child Developmental and Neurobehavioral Factors	0.767	0.580-0.861	0.258-0.965	53.76	88.9	High effect; strong evidence; substantial heterogeneity
Family, Socioeconomic and Perceptual Factors	0.729	0.642-0.800	0.603-0.824	226.32	10.8	High effect; very strong evidence; low heterogeneity

**Figure 9:**
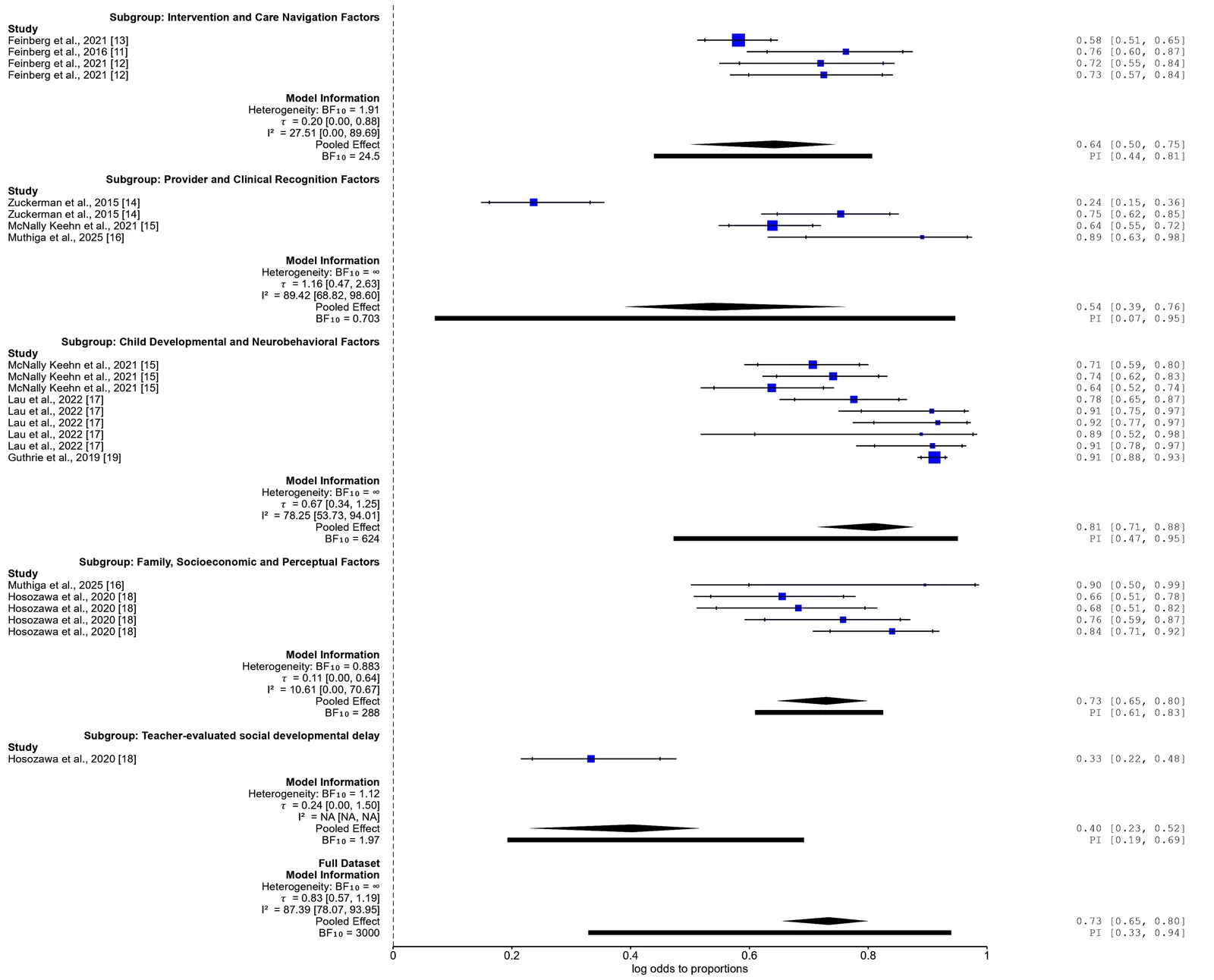
Forest plot of Bayesian subgroup effects by determinant category Forest plot showing pooled Bayesian effects for Intervention and Care Navigation, Provider and Clinical Recognition, Child Developmental and Neurobehavioral, and Family, Socioeconomic and Perceptual Factors. The highest pooled effects were observed for Child Developmental and Neurobehavioral Factors (0.77) and Family, Socioeconomic and Perceptual Factors (0.73), with corresponding 95% credible and prediction intervals.

Meta-Regression Analyses

Meta-regression analyses were conducted to examine whether age group, determinant category, effect measure, and region explained variability in effect sizes. The omnibus tests indicated that determinant category significantly moderated effect sizes (Qₘ=13.483, df=3, p=.004), as did effect measure (Qₘ=7.814, df=2, p=.020). In contrast, neither age group (Qₘ=1.621, df=1, p=.203) nor region (Qₘ=0.563, df=1, p=.453) significantly moderated effect sizes. Thus, the observed heterogeneity in effect sizes appears to be more strongly related to the type of determinant examined and the statistical effect measure used, rather than differences in participant age group or geographic region.

At the coefficient level, the determinant-category contrasts showed that studies examining Child Developmental and Neurobehavioral Factors had significantly larger effect sizes than the omitted reference determinant category (B=1.516, 95% CI: 0.047-2.985, p=.043). Similarly, Family, Socioeconomic and Perceptual Factors were associated with significantly larger effect sizes (B=2.703, 95% CI: 0.661-4.744, p=.009). The contrast for Provider and Clinical Recognition Factors was not statistically significant (B=0.398, 95% CI: −1.340-2.136, p=.653). For effect measure, the aRRR category was associated with significantly smaller effect sizes relative to the omitted reference effect-measure category (B=−2.597, 95% CI: −4.419 to -0.775, p=.005), whereas the contrast for AOR did not reach statistical significance (B=-1.276, 95% CI: -2.751-0.198, p=.090). The coefficient for age group >60 months was not significant (B=0.588, 95% CI: -0.317-1.494, p=.203), consistent with the non-significant omnibus age-group test. Likewise, Africa (Kenya) did not differ significantly from the omitted regional reference category (B=0.683, 95% CI: −1.101-2.467, p=.453). Overall, these findings indicate that determinant category and effect measure were significant sources of between-study variation, whereas age group and region did not provide evidence of systematic moderation of effect sizes (Table 15).

**Table 15:** Meta-regression results examining moderators of effect sizes Qₘ=Omnibus test statistic for moderator effects in meta-regression; df=degrees of freedom; p=p-value. Fixed-effects meta-regression tests were conducted using a z-distribution. The model partially removed one coefficient for Effect Measure and one coefficient for Region because of missing values, collinearity, or empty/missing category combinations. Consequently, coefficient-level findings should be interpreted with caution.

Predictor	Qₘ	df	p-value
Age group	1.621	1	0.203
Determinant Category	13.483	3	0.004
Effect Measure	7.814	2	0.02
Region	0.563	1	0.453

Discussion

Review of the Main Findings

This systematic review identified a broad range of individual, family, socioeconomic, geographic, communication, screening, and health-system determinants of access to ASD diagnostic services. The quantitative synthesis of nine unique studies and 23 effect estimates yielded a pooled proportion of 74.1% (95% CI: 65.8%-81.1%), indicating that approximately three-quarters of the observed diagnostic outcomes represented successful access. However, substantial heterogeneity was evident (I²=88.4%), suggesting that access to ASD diagnosis varies considerably across populations, settings, and diagnostic processes. This finding is consistent with earlier evidence showing that ASD diagnosis is influenced by socioeconomic circumstances, parental recognition, interactions with health and education systems, and geographic availability of services [34].

The high heterogeneity is important because access to ASD diagnosis is not a single event but a multistage process involving recognition of developmental concerns, referral, assessment, and completion of diagnosis. Previous reviews similarly describe the pathway as complex, with delays arising from provider knowledge, unclear referral pathways, service shortages, cost, and communication difficulties [35,36]. Thus, the overall pooled estimate should be interpreted as a summary of a heterogeneous diagnostic process rather than as a universal probability of obtaining an ASD diagnosis.

Outcome Domain and Diagnostic Access

The stratified analysis provides an important refinement of the overall finding. Successful outcomes were highest for diagnostic pathways (89.3%), followed by timely diagnosis (76.3%), and lowest for diagnostic completion (67.1%). In practical terms, families appeared more likely to enter or navigate a diagnostic pathway than to complete the diagnostic process, while timely diagnosis fell between these two outcomes. The borderline subgroup difference (Qₘ=5.98, p=.050) suggests that the stage or aspect of diagnosis being considered may partly explain variation in access.

This pattern is consistent with qualitative evidence describing ASD diagnosis as a prolonged and sometimes fragmented journey. Parents commonly report difficulty knowing where to seek assessment, navigating multiple services, and dealing with prolonged waiting periods even after concerns have been recognized [37]. The distinction between pathway entry and diagnostic completion is therefore important: recognition and referral do not necessarily translate into completed assessment. The particularly high heterogeneity for timely diagnosis (I²=91.2%) further suggests that what constitutes a timely diagnosis varies substantially between studies and settings. This is consistent with findings that age at diagnosis varies considerably and that socioeconomic status, parental concern, interactions with healthcare and education systems, and geographic resources contribute to differences in diagnostic timing [34].

Socioeconomic, Family and Perceptual Factors

Socioeconomic and family-related factors emerged as an important component of diagnostic access. Lower household income, lower parental education, rural residence, limited resources, and reduced ability to navigate services were repeatedly associated with delayed or less equitable access in the narrative evidence. These findings are consistent with the wider literature showing that socioeconomic advantage and greater parental recognition of developmental concerns are associated with earlier diagnosis [34].

The quantitative findings further support the importance of this domain. Family, Socioeconomic and Perceptual Factors had a pooled effect of 74.2%, and the meta-regression showed significantly larger effect sizes for this category relative to the reference category (B=2.703, p=.009). The Bayesian analysis also provided strong evidence for this determinant category (BF=226.32) with relatively low heterogeneity. Taken together, these findings suggest that the social and perceptual environment in which families recognize and respond to developmental concerns may be an important determinant of subsequent access.

This interpretation is also supported by qualitative research showing that families experience financial burdens, long waiting periods, difficulties navigating services, and variable responsiveness from healthcare providers during the diagnostic process [38]. Importantly, these barriers may interact rather than operate independently: families with fewer financial resources may have greater difficulty travelling to specialist services, pursuing private assessment, or repeatedly following up referrals.

Child Developmental and Neurobehavioral Factors

Child developmental and neurobehavioral characteristics were associated with the highest pooled outcome in the frequentist analysis (79.5%) and remained strongly supported in the Bayesian analysis (76.7%; BF=53.76). The meta-regression also indicated significantly larger effect sizes for this category (B=1.516, p=.043). This finding is plausible because the type, severity, and visibility of developmental and behavioral characteristics can influence whether ASD is recognized, suspected, and referred for diagnostic assessment. Earlier evidence has consistently shown that greater symptom severity, developmental impairment, and greater parental concern are associated with earlier ASD identification and diagnosis [34].

This association has also been demonstrated in more recent empirical studies. In a Canadian cohort of 421 preschool children, more advanced language and cognitive skills were associated with later diagnosis, while ASD symptom severity contributed independently to variation in age at diagnosis [38]. Similarly, a population-based surveillance study found that children with lower IQ and developmental regression were identified at younger ages, suggesting that more readily observable developmental differences may prompt earlier recognition [39]. More recent evidence from 801 autistic children likewise found that language delay and developmental regression were associated with earlier diagnosis, whereas cognitive and adaptive delays showed more complex relationships with diagnostic timing [40]. Conversely, children with subtler or less readily recognizable presentations may experience longer diagnostic pathways. A study of high-risk children found that those diagnosed later had more advanced language and adaptive skills and milder ASD symptoms than children diagnosed at 18-24 months [41]. Similarly, evidence from a large parent-reported study suggests that clinical signs that are more easily recognized as developmental concerns are more strongly associated with earlier diagnosis, whereas children without early communication deficits may experience delayed diagnosis [5]. Importantly, recent evidence from Kenya also demonstrates that specific developmental and neurobehavioral characteristics - including delayed walking, attention-deficit hyperactivity disorder (ADHD), and intellectual developmental disorder - can be associated with diagnostic delay, highlighting the relevance of child-level presentation within lower-resource diagnostic systems [16].

The findings also reinforce the importance of systematic developmental surveillance and screening. A study concluded that systematic monitoring and ASD-specific screening during early childhood can facilitate earlier identification, although screening alone does not eliminate downstream barriers to diagnostic assessment [42]. Thus, identifying developmental concerns is only the first step; effective referral and diagnostic capacity are required to convert recognition into completed diagnosis.

Health-System, Provider and Navigation Factors

The review identified health-system capacity, provider recognition, referral coordination, specialist availability, and navigation as important determinants of access. These findings are consistent with broader reviews identifying shortages of services, limited provider awareness, unclear referral pathways, and inadequate coordination as major barriers to ASD healthcare access [35].

The evidence concerning Intervention and Care Navigation Factors was particularly relevant. Family navigation was associated with improved access in several quantitative studies, while the pooled estimate for this category was 68.0%. External evidence similarly suggests that navigation can help families overcome practical and system-level barriers. For example, a navigation study among disadvantaged families with children screening positive for ASD found that families commonly experienced multiple barriers to referral and evaluation, with navigation specifically used to address these obstacles.

However, provider and clinical recognition factors showed greater uncertainty. Their pooled effect was not statistically significant in the frequentist analysis, the Bayesian evidence for inclusion was weak (BF=0.684), and heterogeneity was high. This suggests that provider-related factors may operate differently depending on the healthcare system, provider expertise, clinical presentation, and referral context rather than having a uniform effect across settings.

Geographic and Communication Inequities

Although geographic region did not significantly moderate effect sizes (Qₘ=0.563, p=.453), the narrative synthesis identified important geographic barriers. Rural and underserved populations may face longer travel distances, fewer specialists, fragmented referral pathways, and longer waiting periods. These barriers are consistent with evidence that geographic variation in diagnostic age reflects differences in community resources and healthcare systems [21].

Similarly, communication emerged as an important pathway through which inequities may occur. Effective communication between parents, teachers, primary-care providers, and specialists facilitates recognition and referral, whereas language barriers or dismissal of parental concerns can delay diagnosis. Recent qualitative syntheses have emphasized the importance of healthcare professionals listening to and validating parental concerns and providing clear information throughout the diagnostic process [37].

Screening and Early Identification

The narrative evidence indicates that screening can improve early identification, but its effectiveness depends on implementation and follow-up. This is consistent with recommendations emphasizing systematic developmental surveillance and ASD screening during early childhood [42]. However, screening should not be interpreted as sufficient on its own. A positive screen must be followed by accessible referral, specialist assessment, and diagnostic completion.

This distinction helps explain why the present review found a relatively high pooled outcome for diagnostic pathways but a lower outcome for diagnostic completion. A health system may successfully identify children at risk and initiate referrals while still failing to provide sufficient capacity to complete diagnostic assessments. Thus, improving screening without strengthening downstream diagnostic capacity may simply shift the point at which families encounter delays.

Interpretation of Heterogeneity and Meta-Regression

The substantial overall heterogeneity (I²=88.4%) indicates that diagnostic access is highly context-dependent. Importantly, the meta-regression identified determinant category and effect measure, rather than age group or geographic region, as significant moderators. Determinant category therefore appears to capture meaningful differences in the mechanisms through which access is achieved or constrained.

The significant effect-measure moderation should nevertheless be interpreted cautiously. Different measures - HR, AOR, aRRR and APR - represent different statistical quantities and are not necessarily directly interchangeable in substantive meaning. The observed moderation may therefore partly reflect methodological differences between studies rather than true differences in diagnostic access. The partial removal of coefficients for effect measure and region also indicates sparse cells and should further discourage over-interpretation of individual contrasts.

Strengths and limitations

A major strength of this review is its multidimensional assessment of ASD diagnostic access, incorporating diagnostic completion, pathways, and timely diagnosis, alongside complementary frequentist and Bayesian analyses. However, several limitations should be considered. Only nine unique studies contributed quantitative data, limiting the precision of some subgroup and meta-regression estimates. Differences in study designs, populations, outcome definitions, and effect measures contributed to substantial heterogeneity. Multiple effect measures also mean that pooled estimates should be interpreted as standardized summary estimates rather than directly equivalent probabilities. Sparse or missing data resulted in the removal of some meta-regression coefficients, limiting interpretation of certain moderators. Although the search was conducted through December 2024, literature published after this period was not included and may contain additional evidence relevant to the findings of this review. Finally, the evidence is predominantly from high-income settings, with limited evidence from Africa, restricting generalizability to low-resource contexts.

Implications for practice and research

The findings suggest that improving ASD diagnostic access requires interventions across the entire diagnostic pathway, rather than focusing exclusively on screening. Health systems should strengthen developmental surveillance, referral coordination, provider training, family navigation, specialist capacity, and mechanisms for tracking children from referral to completed assessment. Navigation approaches may be particularly valuable for disadvantaged families facing multiple simultaneous barriers [12].

Future research should prioritize longitudinal studies that measure specific stages of the diagnostic pathway separately and use standardized definitions of diagnostic delay and completion. More evidence is also needed from low- and middle-income countries, where shortages of specialists and diagnostic infrastructure may substantially alter the pathway. Recent evidence from Kenya, for example, demonstrates that children may encounter multiple healthcare contacts before diagnosis, with substantial delays between caregiver recognition and diagnostic confirmation [16].

## Conclusions

Overall, this review demonstrates that access to ASD diagnostic services is substantial but highly variable, with approximately three-quarters of observed outcomes representing successful access. Families are most likely to successfully navigate diagnostic pathways and least likely to complete the diagnostic process, while timely diagnosis shows particularly large variation across studies. The findings indicate that family and socioeconomic circumstances, child developmental characteristics, provider recognition, and health-system organization interact to shape diagnostic access. Improving access therefore requires coordinated interventions that extend beyond screening to ensure that children identified as potentially autistic can progress efficiently from recognition and referral through to completed and timely diagnosis.

## Appendices

The Search Strategy is presented as a supplementary table.

**Table 16:** Supplementary Table S1. Database search strategies

Database	Search strategy
MEDLINE/PubMed	("Autism Spectrum Disorder"[Mesh] OR autism[tiab] OR ASD[tiab] OR autistic[tiab]) AND (diagnosis[tiab] OR "diagnostic services"[tiab] OR screening[tiab] OR "intervention services"[tiab] OR "treatment services"[tiab] OR "healthcare access"[tiab] OR "service availability"[tiab] OR "service utilization"[tiab] OR workforce[tiab] OR specialist*[tiab]) AND ("wait time*"[tiab] OR "waiting time*"[tiab] OR "diagnostic delay*"[tiab] OR "delay to diagnosis"[tiab] OR "age at diagnosis"[tiab] OR timeliness[tiab] OR "access delay*"[tiab])
Embase	(autism OR autistic OR "autism spectrum disorder" OR ASD) AND (diagnosis OR "diagnostic services" OR screening OR "intervention services" OR "treatment services" OR "healthcare access" OR "service availability" OR "service utilization" OR workforce OR specialist*) AND ("wait time*" OR "waiting time*" OR "diagnostic delay*" OR "delay to diagnosis" OR "age at diagnosis" OR timeliness OR "access delay*")
Scopus	TITLE-ABS-KEY(autism OR autistic OR "autism spectrum disorder" OR ASD) AND TITLE-ABS-KEY(diagnosis OR "diagnostic services" OR screening OR "intervention services" OR "treatment services" OR "healthcare access" OR "service availability" OR "service utilization" OR workforce OR specialist*) AND TITLE-ABS-KEY("wait time*" OR "waiting time*" OR "diagnostic delay*" OR "delay to diagnosis" OR "age at diagnosis" OR timeliness OR "access delay*")
Web of Science	TS=(autism OR autistic OR "autism spectrum disorder" OR ASD) AND TS=(diagnosis OR "diagnostic services" OR screening OR "intervention services" OR "treatment services" OR "healthcare access" OR "service availability" OR "service utilization" OR workforce OR specialist*) AND TS=("wait time*" OR "waiting time*" OR "diagnostic delay*" OR "delay to diagnosis" OR "age at diagnosis" OR timeliness OR "access delay*")
Grey literature	Targeted searches using combinations of autism, "autism spectrum disorder", diagnosis, "diagnostic services", "healthcare access", "service availability", "diagnostic delay", "age at diagnosis", timeliness, workforce, and specialist. Reference lists of included studies and relevant reviews were also screened.
